# AI-enhanced flexible ECG patch for accurate heart disease diagnosis, optimal wear positioning, and interactive medical consultation

**DOI:** 10.1093/nsr/nwaf425

**Published:** 2025-10-11

**Authors:** Xiaojiang Huang, Youlin Yuan, Jiming Liu, Jun He, Yunxiang Shi, Shuai Gao, Jun Wu, Xingjie Xu, Huiqing Zhang, Peng Li, Yao Yao, Wei Huang

**Affiliations:** State Key Laboratory of Flexible Electronics (LoFE) & Institute of Flexible Electronics (IFE), Northwestern Polytechnical University, Xi’an 710072, China; School of Mechanics and Transportation Engineering, Northwestern Polytechnical University, Xi’an 710072, China; Queen Mary University of London Engineering School, Northwestern Polytechnical University, Xi’an 710072, China; School of Mechanical Engineering, Xi’an Jiaotong University, Xi’an 710049, China; China Ordnance Industry Group-machine-environment Key Laboratory, Institute for Hygiene of Ordnance Industry, Xi’an 710065, China; State Key Laboratory of Flexible Electronics (LoFE) & Institute of Flexible Electronics (IFE), Northwestern Polytechnical University, Xi’an 710072, China; State Key Laboratory of Flexible Electronics (LoFE) & Institute of Flexible Electronics (IFE), Northwestern Polytechnical University, Xi’an 710072, China; State Key Laboratory of Flexible Electronics (LoFE) & Institute of Flexible Electronics (IFE), Northwestern Polytechnical University, Xi’an 710072, China; State Key Laboratory of Flexible Electronics (LoFE) & Institute of Flexible Electronics (IFE), Northwestern Polytechnical University, Xi’an 710072, China; State Key Laboratory of Flexible Electronics (LoFE) & Institute of Flexible Electronics (IFE), Northwestern Polytechnical University, Xi’an 710072, China; Sanhang Institute for Brain Science and Technology, School of Medical Research, Northwestern Polytechnical University, Xi’an 710072, China; State Key Laboratory of Flexible Electronics (LoFE) & Institute of Flexible Electronics (IFE), Northwestern Polytechnical University, Xi’an 710072, China; State Key Laboratory of Flexible Electronics (LoFE) & Institute of Flexible Electronics (IFE), Northwestern Polytechnical University, Xi’an 710072, China; School of Mechanics and Transportation Engineering, Northwestern Polytechnical University, Xi’an 710072, China; State Key Laboratory of Flexible Electronics (LoFE) & Institute of Flexible Electronics (IFE), Northwestern Polytechnical University, Xi’an 710072, China

**Keywords:** electrocardiogram, interpretable artificial intelligence, wearable device, health monitoring

## Abstract

Continuous and reliable electrocardiogram (ECG) monitoring is crucial for the early diagnosis and intervention of heart diseases, which remain a leading threat to global health and mortality. Traditional ECG devices are often bulky, complex, and require hospital visits, limiting their practicality for daily use. To overcome these challenges, we have developed a wireless, flexible, and user-friendly ECG monitoring system integrated with advanced artificial intelligence (AI) capabilities. Our innovative ECG patch features an island-and-bridge serpentine structure, offering strain insensitivity of up to 100%, robust adhesion (7.6 kPa), and a high signal-to-noise ratio (28 dB). The accompanying mobile application leverages the interpretable attention transformer (IAT) model for heart disease diagnosis with up to 98% accuracy, a generative adversarial network (GAN) combined with convolutional neural networks (CNNs) and gated recurrent units (GRUs) for wear positioning correction with 85% accuracy, and GPT-based consultations with sub-second response times. This system enables real-time diagnosis, accurate wear positioning, and personalized medical advice, effectively bridging the gap between hospital care and at-home monitoring. Our work enhances accessibility to cardiac care, promotes early detection, and reduces the burden on healthcare systems.

## Teaser

This AI-enhanced flexible ECG patch with an island-and-bridge serpentine structure enables accurate heart disease diagnosis and correct wearing positioning, and provides interactive medical advice for home monitoring.

## INTRODUCTION

Heart disease is the leading global cause of death, costing >$200 billion yearly in healthcare, medications, and premature death–related productivity losses [1–3]. Early diagnosis and intervention are crucial for lowering mortality risk and enhancing cardiovascular health through electrocardiogram (ECG) monitoring [4–7]. ECG monitoring stands out as an effective method for evaluating heart health [8,9]. However, conventional ECG devices are often bulky, complex, and require hospital visits for expert interpretation. This reliance on clinical settings impedes timely diagnosis and precise treatment, highlighting the urgent need for advanced wearable systems that combine long-term accurate ECG monitoring with interpretable AI diagnostics and personalized medical consultations [10–12].

Flexible electronics have emerged as a promising solution for wearable devices due to their ability to conform to the body’s contours, thereby enhancing comfort, wearability, and user experience while maintaining superior signal quality [13–16]. Despite these advantages, motion-induced interference remains a significant challenge, as physical activities such as running or jumping can introduce noise and artifacts that distort ECG waveforms, thereby compromising diagnostic accuracy [17]. Recent advancements in wearable ECG technology have focused on mitigating these motion artifacts through both hardware and software innovations. Hardware-based strategies include the development of crack-based sensors [18], serpentine structures [19], stress dispersion [20], and localized hardening [21]. Concurrently, software-based approaches such as adaptive filtering [22], wavelet transform [23], and machine learning [24] have been employed to enhance signal fidelity. Nevertheless, even with high-quality ECG signals, non-experts often struggle to assess heart health and correctly position devices, highlighting the need for AI solutions that support diagnosis, guide placement, and enable medical consultations. These features can improve the usability and reliability of wearable ECG devices for everyone.

Various methodologies have been explored to diagnose conditions such as atrial fibrillation (AF), myocardial infarction (MI), and other arrhythmias. Deep learning models, particularly convolutional neural networks (CNNs) and recurrent neural networks (RNNs) like long short-term memory (LSTM) networks or transformers, have demonstrated significant potential in extracting pertinent features from raw ECG data without the need for manual feature engineering [25]. These models facilitate personalized health recommendations based on individual health metrics, including ECG signals, blood pressure, and heart rate, thereby aiding in disease diagnosis [26]. However, the ‘black-box’ nature of many deep learning algorithms means their decision-making processes remain unclear. This lack of interpretability poses a major obstacle, as it undermines clinical trust and makes it more difficult for non-expert patients to understand their diagnoses. In fact, only a small fraction of current AI-assisted ECG systems provides truly interpretable results, limiting the broader adoption of these technologies in healthcare [27–29]. Additionally, while portable ECG devices offer the convenience of continuous heart health monitoring, improper device placement may lead to inaccurate readings and potential misdiagnoses. Consequently, implementing AI-based automated wear positioning correction is essential to ensure the reliability of ECG data.

In this study, we have designed a wireless, wearable, and intelligent ECG monitoring system designed for user-friendliness and minimal motion interference. Our system integrates AI-assisted heart disease diagnosis, precise wear positioning correction, and interactive medical consultations. The strain-insensitive ECG patch, capable of withstanding up to 100% strain, features robust adhesion (7.6 kPa) and a high signal-to-noise ratio (28 dB). Both experimental data and simulations validate that the ‘island-and-bridge’ structural design ensures high-precision ECG data acquisition even under dynamic conditions. The accompanying mobile application delivers accurate heart rate measurements (95% accuracy), heart rate variability analysis, and a sophisticated generative adversarial network (GAN) algorithm for wear positioning assessment (85% accuracy). Additionally, it integrates an interpretable attention transformer (IAT) algorithm for heart disease diagnosis, achieving up to 98% accuracy. Furthermore, the integration of GPT-based consultations provides real-time medical advice within 1 second. This comprehensive ECG monitoring system enables real-time AI-assisted diagnosis, precise wear positioning assessment, and personalized medical consultations, facilitating long-term home-based ECG monitoring. Consequently, it supports early diagnosis and effective prevention of heart diseases, making advanced cardiac care accessible even to users without professional medical expertise.

## RESULTS AND DISCUSSION

### Our AI-assisted flexible ECG patch provides continuous, accurate heart monitoring and intelligent diagnostic support

**Figure 1.**
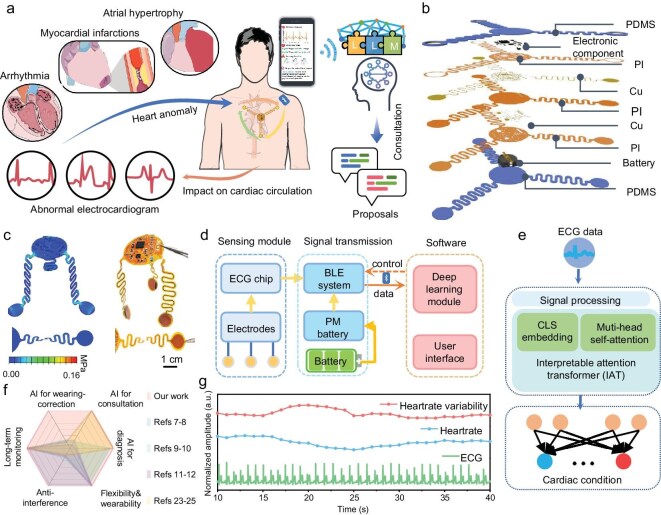
Wireless heart monitoring system with AI assistance. (a) AI-assisted electrocardiogram (ECG) patch for heart disease diagnosis, wearing-position correction, and GPT consultation with large language model (LLM). (b) The ECG patch construction. (c) Finite element simulation and the physical product. (d) The wireless ECG system architecture diagram. (e) Interpretable attention transformer (IAT) for heart disease diagnosis. (f) Comparison with existing literature. (g) ECG data from hospital patients.

To develop a continuous, reliable, and intelligent heart monitoring system, we engineered an AI-assisted flexible ECG system that integrates advanced hardware and sophisticated software functionalities (Fig. 1a). The flexible ECG patch with a reliable serpentine structure, ensures precise and dependable ECG signal acquisition even during dynamic activities (Fig. 1b).

To evaluate the mechanical performance of the serpentine design, we conducted finite element analysis (FEA). The analysis revealed that external forces are predominantly concentrated on the serpentine sections, which absorb these forces through deformation. This design effectively minimizes the impact of mechanical stress on overall signal acquisition, maintaining high signal integrity under various conditions (Fig. 1c).

The flexible ECG patch comprises two primary modules: the ECG signal acquisition module and the data transmission module (Fig. 1d). The ECG acquisition module includes an ECG chip that collects biopotential signals from the skin surface and converts differential signals into a single-ended analog output. This analog signal is then digitized by the main control chip through an analog-to-digital converter (ADC). The resulting digital data is transmitted via Bluetooth to a mobile application, where it is visualized, analyzed, and utilized for further diagnostic processes.

To enhance disease recognition accuracy automatically for users, we designed a novel deep learning algorithm termed interpretable attention transformer (IAT). The IAT can capture long-range dependencies within sequential signals, such as ECG signals, using self-attention mechanisms. This algorithm has demonstrated significant potential in processing the complex data associated with disease diagnosis. Moreover, one of the standout features of the IAT algorithm is its interpretability, allowing us to understand which parts of the model focus on during the learning process, which is crucial for building trust with non-medical users and ensuring the reliability of the disease classification results (Fig. 1e).

We have compared our AI-assisted ECG monitoring system with existing ECG devices and found that our system offers significant advancements in AI-assisted diagnosis, wear positioning correction, and medical consultation capabilities (Fig. 1f). The integration of these AI features not only improves diagnostic accuracy but also enhances user experience by providing real-time feedback on device placement and presenting interactive medical consultations.

Finally, our flexible ECG monitoring system facilitates continuous and reliable home-based heart monitoring for adults (Fig. 1g and Video S1). This capability reduces the burden on caregivers and healthcare providers while increasing monitoring efficiency and accessibility. The system’s comprehensive features support early diagnosis and effective prevention of heart diseases, making advanced cardiac care attainable for individuals without professional medical expertise.

### Our flexible ECG patch demonstrates superior structural integrity and functionality under dynamic conditions

**Figure 2.**
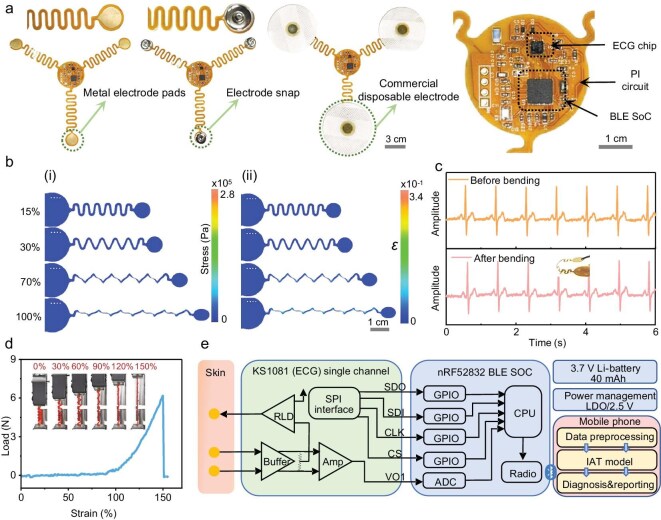
Structure and function of ECG patch. (a) ECG patch with enlarged views of the core circuit area. (b) Finite element simulation of (i) stress and (ii) strain under uniaxial tensile loading. (c) Bending test of the ECG patch. (d) Mechanical strength evaluation of the patch. (e) Hardware architecture and software framework diagram.

To enhance the resilience of the ECG patch against motion-induced strain, we designed the patch with island-like features and serpentine structures. We incorporated soldered electrode connectors for seamless attachment to commercial disposable electrodes (Fig. 2a and Fig. S1). The ECG electrodes comprise three dry electrodes evenly distributed in three directions, interconnected to the central circuit through serpentine wiring. This configuration enables the flexible ECG patch to detect minute electrical signals, which are subsequently transmitted to the Bluetooth system-on-chip (SoC) for 50 Hz power-line frequency filtering. The electrode outperformed commercial Ag/AgCl electrodes in all aspects, showing lower impedance, better adhesion, reduced motion artifacts, stable long-term performance, and superior sweat resistance, demonstrating its suitability for dynamic ECG monitoring (Figs S2 and S3).

We performed finite element simulations to evaluate the mechanical properties of the serpentine structure. The simulations revealed that stress concentrates primarily along the serpentine wires when external forces are applied, with the maximum stress localized at the serpentine bends (Fig. 2b). This design effectively minimizes impedance variations and component stress, maintaining a high signal-to-noise ratio of 28 dB even when the device is bent or stretched (Fig. 2c). The serpentine architecture, by improving structural compliance, reduces peak stress by 8% and peak strain by 58% compared to non-serpentine designs, thereby enhancing reliability (Fig. S4).

To validate the mechanical performance experimentally, we conducted force-resistance tests. We observed that the resistance changes by only 0.5% when the strain ranges from 0% to 100%, and remains within −0.1% to 0.2% over 300 stretching cycles (Fig. S5). These results confirm the structure’s low strain sensitivity, with a gauge factor of 0.005. Additionally, the device withstands stretching up to 150% of its original length without any performance degradation (Fig. 2d), demonstrating its exceptional flexibility and resilience. The electrodes are arranged in an equilateral triangular configuration to balance aesthetic design and mechanical robustness, addressing user experience and functional reliability, whereas asymmetric layouts would induce uneven stress, potentially distorting ECG signals or damaging the patch (Fig. S6).

We constructed the ECG monitoring system by integrating the flexible ECG patch with a mobile application (Fig. 2e). The patch features three electrodes configured in a single-lead setup: two electrodes capture small differential signals, while the third serves as a right-leg drive circuit to effectively mitigate common-mode interference. The ECG amplifier, equipped with an SPI interface, connects to an analog front-end chip. The SoC processes the analog signal from the ECG chip before transmitting the data via Bluetooth to a mobile device for rapid analysis and disease recognition (Fig. S7).

The mobile application facilitates real-time visualization of the collected ECG data, which we process using the Pan-Tompkins algorithm to extract vital parameters such as heart rate and heart rate variability. These metrics are updated every 6 seconds and displayed in real-time. Furthermore, the mobile application employs a generative adversarial network (GAN) and an interpretable attention transformer (IAT) model to classify ECG signals from various wearable positions and identify different types of heart diseases.

### Flexible ECG patch accurately detects cardiac anomalies and maintains signal integrity during motion

**Figure 3.**
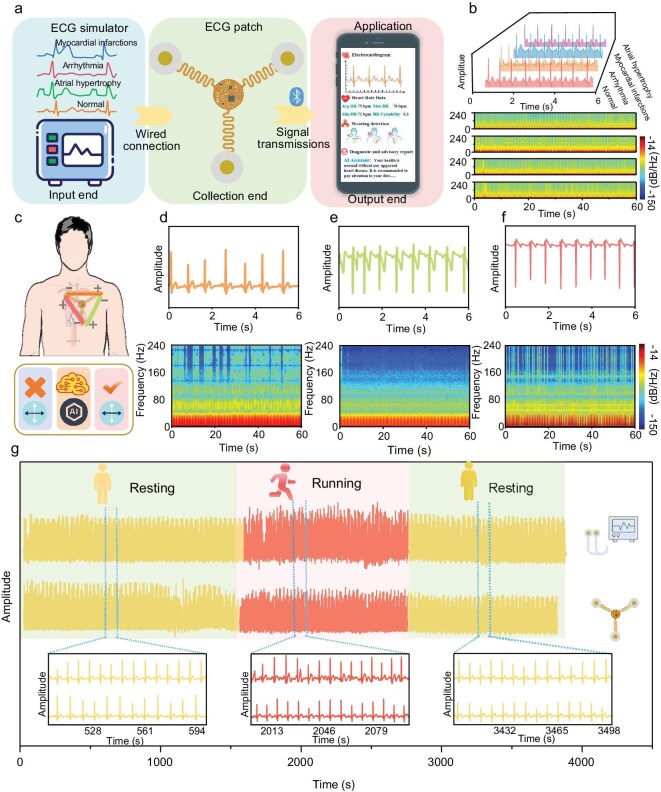
On-body evaluation of wearable ECG patch for heart disease diagnosis, wearing-position correction, and comparison with a commercial ECG device. (a) Overview of the flow of ECG patch system operation. (b) Four types of ECG signal tests and corresponding instantaneous frequency diagrams. Schematic illustration of (c) position correction and (d–f) test results of three typical positions. (g) Comparison of real-time monitoring between the commercial ECG device and our ECG patch.

To evaluate the flexible ECG patch’s capability to monitor various cardiac anomalies, we utilized a commercial ECG simulator to generate a diverse range of abnormal ECG patterns, including myocardial infarction, arrhythmia, and atrial hypertrophy. These simulated ECG signals were input into the ECG patch, with the resulting data recorded on a mobile device (Fig. 3a). The analysis of the waveform and time-frequency characteristics from the abnormal ECG signals demonstrated that the flexible ECG patch effectively preserves signal integrity, highlighting its robust monitoring capabilities and potential for comprehensive ECG signal analysis (Fig. 3b). The results reveal that the ECG patch can reliably identify and differentiate between various cardiac anomalies, emphasizing its utility in clinical diagnostics.

To interpret the ECG data, we conducted waveform analysis, which involves examining the shape, amplitude, and duration of ECG waves. For instance, shifts in the ST segment indicate myocardial infarction, variations in the R-R intervals suggest arrhythmias, and alterations in the P wave morphology signify atrial hypertrophy. Additionally, we employed a time-frequency analysis algorithm to assess how the frequency components of the ECG signal change over time. This method enhances detection accuracy by identifying subtle changes that traditional waveform analysis might miss. We observed that the time-frequency analysis algorithm provided valuable insights into the device’s ability to accurately identify and differentiate between various cardiac events, thereby enhancing overall diagnostic precision.

However, we recognized that ECG signals are highly sensitive to electrode placement relative to the heart. Due to the symmetrical design of the ECG patch and the potential lack of professional knowledge among users, improper wearing positions can occur, leading to distorted ECG waveforms (Fig. 3c). We observed that incorrect wearing positions caused significant distortions in the captured ECG signals, including baseline shifts, amplitude variations, and waveform shape alterations (Fig. 3d–f). These distortions can obscure or exaggerate certain cardiac events, potentially resulting in misdiagnosis or missed diagnoses. The results highlight that accurate and reliable ECG monitoring necessitates the correction of wearing positions to ensure optimal signal quality.

To overcome the issue of improper wearing positions, we employed deep learning techniques to classify ECG waveforms from three different orientations, facilitating the accurate determination of the correct wearing position (Fig. S8). The deep learning models could efficiently classify the wearing positions based on noticeable differences in the ECG waveforms. The results indicate that integrating deep learning models for wear positioning correction significantly enhances the reliability of ECG recordings, ensuring accurate diagnosis.

Furthermore, to assess the flexible ECG patch’s resistance to motion interference, we conducted comparative studies with traditional wired ECG monitoring devices during both exercise and rest periods. We observed that the flexible ECG patch significantly reduces motion-induced interference during physical activity (Fig. 3g). To quantify signal quality, we measured baseline drift (normalized units) and SNR: our system showed smaller drift (0.0165 total amplitude vs. 0.1449) and higher SNR (27.58 dB vs. 21.60 dB in dynamic conditions; 28.82 dB vs. 27.00 dB at rest) than the commercial device, surpassing it in both scenarios.

To validate the claim of ‘no performance degradation’ under deformation, we conducted experiments on mechanical loading and ECG signal collection. Under 150% tensile strain and 180° torsional deformation, the device showed a cross-correlation coefficient of ≥0.92, an SNR decrease of <0.05 dB, and a key ECG metrics retention rate of >98%. These results confirm that the serpentine structure’s flexibility ensures functional stability (Figs S9 and S10). The serpentine connections in the patch allow it to stretch and flex, accommodating the body’s movements during exercise. This design minimizes motion-induced disturbances to the core circuitry and eliminates issues related to poor contact at connection points. The results indicate that our flexible ECG patch maintains high signal integrity during physical activities, improving the accuracy and reliability of ECG monitoring compared to traditional devices. This innovation not only enhances ECG monitoring during exercise but also serves as a reference for the development of more advanced wearable devices.

### AI-assisted wearable ECG patch enables high-accuracy heart disease diagnosis via deep learning integration

**Figure 4.**
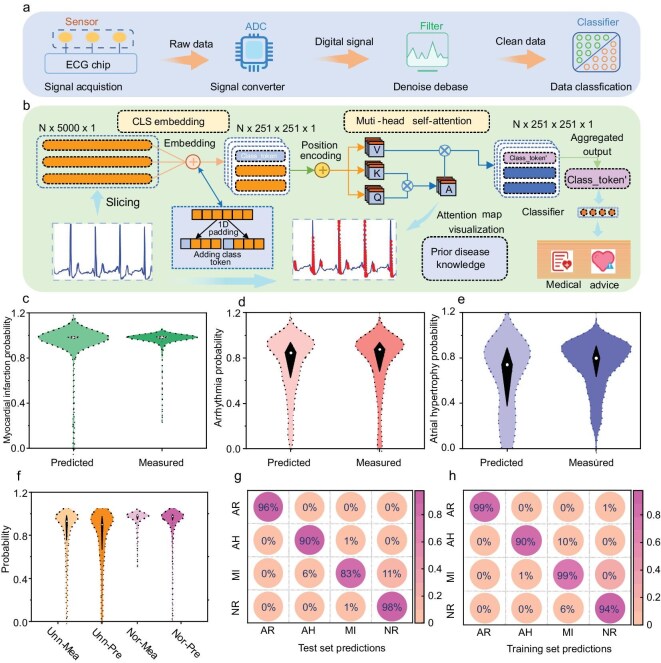
Deep learning for heart disease diagnosis. (a) Mechanism of disease diagnosis and (b) interpretable attention transformer (IAT) for ECG signal classification. Violin plots showing the predicted and measured probability distributions for (c–e) three heart diseases and (f) normal and abnormal cases. (g and h) Confusion matrix for the training and test set, respectively, for arrhythmias (AR), atrial hypertrophy (AH), myocardial infarction (MI), and normal (NR).

To facilitate self-diagnosis of heart conditions for individuals without professional medical expertise, we integrated high-precision, feature-rich ECG signals with a well-trained neural network. We developed a wearable ECG system composed of three main components, i.e. an ECG patch acquisition module, a preprocessing module, and a deep learning module (Fig. 4a). The ECG patch acquisition module includes semi-dry ECG electrodes, a signal converter, and an analog-to-digital converter. These electrodes are engineered for comfortable skin adhesion, ensuring the capture of weak ECG signals with high fidelity. The signal converter amplifies these signals, enhancing the signal-to-noise ratio while minimizing additional noise, before converting the amplified signals into voltage data for further analysis.

We implemented a preprocessing module to ensure the quality of ECG signals through employing algorithms for denoising, baseline correction, segmentation, and classification. These preprocessing steps are crucial for minimizing variability and providing high-quality input to the deep learning model. We propose an interpretable attention transformer (IAT) for ECG signal classification. The ECG signals are first segmented into slices, followed by convolutional operations to extract local features, and a class token (CLS) is appended to each segment. These segments, along with the CLS token, are then processed by a multi-head self-attention encoder, which outputs both the encoded representations and the attention score matrix. The CLS token serves as a global representation of the ECG signal and is projected into logits for classification, effectively aggregating information from local regions through the attention mechanism. Furthermore, the attention score matrix enhances interpretability by highlighting high-attention regions in the ECG signal, which is consistent with clinically significant areas that physicians focus on during diagnosis. This interpretability aligns the model’s decision-making process with human reasoning, making the IAT not only effective for ECG classification but also inherently explainable (Fig. 4b). We developed the IAT architecture instead of traditional Bi-LSTM or CNN models due to its superior interpretability, which enables a clearer understanding of the model’s decision-making process and highlights key ECG features essential for accurate diagnosis [30–32]. The multi-head attention mechanism in the IAT model enables the simultaneous parsing of pathological and artifact-related features, addressing the critical challenge of distinguishing heart disease from misalignment-induced waveform anomalies (Figs S11 and S12).

We conducted experiments with 52 participants, capturing ECG signals using the wearable patch and cross-validating them against measurements from a medical-grade system to ensure data reliability. The resulting dataset comprises over 2000 ECG sets, partitioned into 80% for training and 20% for testing. The trained IAT model effectively discriminates between normal and abnormal ECG signals, as evidenced by the violin plots in Fig. 4c–f, which illustrate the distribution of prediction and measurement probabilities for atrial hypertrophy, myocardial infarction, and arrhythmias. These violin plots reveal data distribution peaks above 80%, indicating high accuracy. Specifically, the test set yields average accuracy rates of 96% for arrhythmias, 90% for atrial hypertrophy, and 83% for myocardial infarction, while the training dataset achieves 99%, 90%, and 99%, respectively, demonstrating the model’s robustness and generalizability in real-world applications (Fig. 4g and h). The IAT model outperforms conventional models like LSTM and Bi-LSTM in disease diagnosis, achieving an average accuracy of 91%, which is 6% higher than the Bi-LSTM model (average accuracy of 85%) and significantly higher than the LSTM model (average accuracy of 15%) that uses raw, unprocessed ECG signals (Fig. S13).

The distribution of normal and abnormal ECG predictions, shown in Fig. 4f, predominantly exceeds 80% accuracy, underscoring the system’s potential for precise cardiac diagnosis. Additionally, the confusion matrix depicted in Fig. 4g and h highlights the model’s superior performance. These results indicate that our AI-assisted wearable ECG patch, combined with interpretable attention transformer, provides reliable and accurate heart disease diagnosis.

This comprehensive study demonstrates the robust capability of the AI-assisted wearable ECG patch in assessing and monitoring heart health. The system’s integration into daily life could significantly impact healthcare by enabling early detection and self-diagnosis, thereby enhancing preventative measures and reducing the burden on healthcare professionals.

### AI-assisted wearable ECG patch enables effective home-based heart disease diagnosis with enhanced usability and accessibility

**Figure 5.**
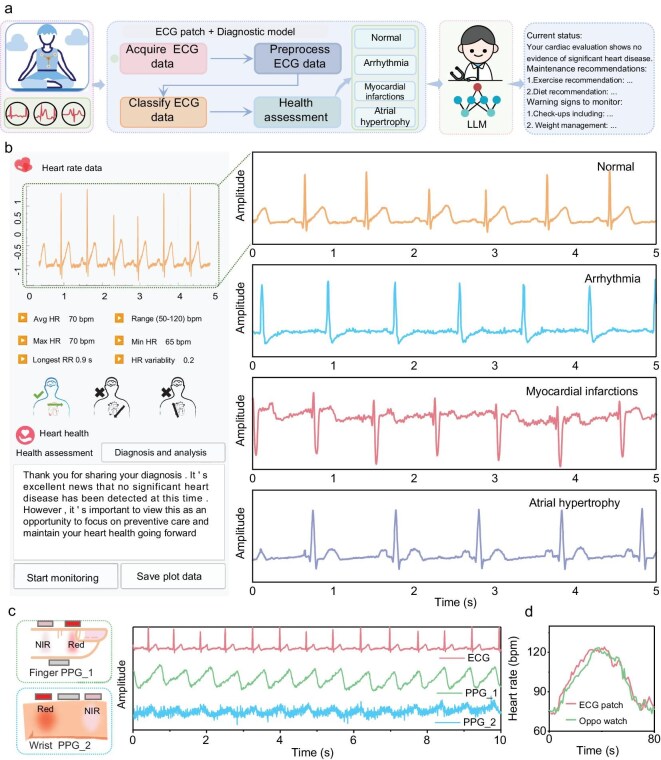
Household diagnosis of AI-assisted wearable ECG patch. (a) Schematic diagram of household diagnosis. (b) Mobile interface and ECG results of hospital patients. (c) Signal-to-noise ratio (SNR) comparison among the ECG patch, wrist photoplethysmography (PPG), and finger PPG. (d) Heart rate comparison between the ECG patch and the Oppo watch in a dynamic environment.

To provide a remote, intelligent, and user-friendly cardiac monitoring solution, we developed an AI-assisted wearable ECG patch that surpasses traditional commercial and hospital-based ECG systems (Fig. 5a).

To achieve portability, we designed a conformal, flexible ECG patch for wireless, long-term household monitoring. Such a design liberates users from the constraints of wired connections typical in traditional ECG devices, allowing uninterrupted monitoring in the comfort of their home environment and eliminating the need for frequent hospital visits. The results indicate that the ECG patch enhances user convenience and facilitates continuous heart monitoring outside clinical settings.

To enable real-time monitoring and diagnosis, we integrated sophisticated algorithms, including the Pan-Tompkins algorithm, to extract vital parameters such as heart rate and heart rate variability from the collected ECG data. Additionally, the IAT deep learning model within the mobile application classifies heart diseases, providing immediate insights and alerts. We observed that this real-time capability allows for the prompt detection of potential cardiac issues, which is crucial for timely intervention. The results indicate that our system supports effective and immediate heart condition diagnosis, enhancing patient outcomes.

To ensure ease of use, we developed a user-friendly graphical interface that presents real-time ECG waveforms, heart rate, heart rate variability, and personalized health advice (Fig. 5b and Fig. S14). This intuitive design allows users without medical expertise to easily interpret their data and take necessary action. The results indicate that the system’s interface improves accessibility and empowers users to engage in proactive health management.

To provide remote access to medical advice, we integrated large language models, such as ChatGPT and DeepSeek, into the system via API, offering users instant health advice, including dietary recommendations, exercise guidance, and medical suggestions. This integration facilitates immediate access to professional medical insights from the comfort of the users home (Fig. S15). Moreover, the ECG patch can acquire high SNR ECG signals. These signals are close to the gold-standard finger photoplethysmography (PPG) signals and have a higher SNR than wrist PPG signals. This is beneficial for accurate heart rate monitoring during sports (Fig. 5c and d). The results indicate that our system not only monitors heart health but also supports comprehensive health management through personalized advice from monitoring information.

These integrated features reduce the frequency of hospital visits by providing real-time ECG monitoring and convenient access to professional medical insights from home. The diagnostic results from ECG monitoring are transmitted to an AI-assisted virtual doctor, which offers personalized health recommendations, including dietary advice, exercise guidance, and medical suggestions (Figs S17–S19). These functionalities significantly alleviate the burden on healthcare facilities by facilitating early detection and self-diagnosis.

The results demonstrate that our AI-assisted wearable ECG patch provides prompt and effective health monitoring and guidance, thereby enhancing patient care and alleviating the burden on healthcare systems.

### Intelligent ECG software enables real-time visualization, automated analysis, and personalized health recommendations

**Figure 6.**
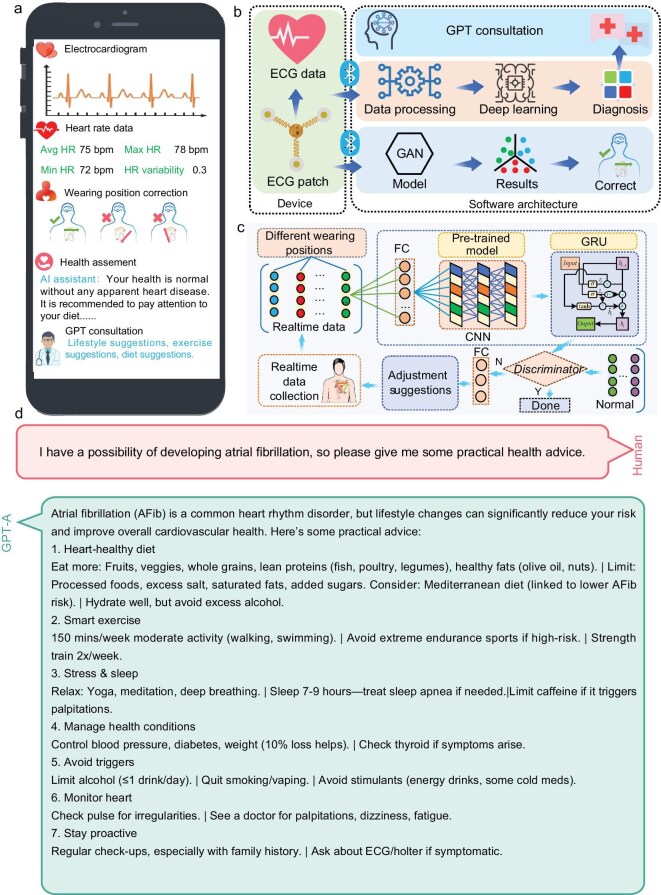
Illustration of the app. (a) The mobile app interface includes four sections: real-time ECG, heart rate data, wearing-position correction, and health assessment and GPT consultation. (b) Internal software architecture. Wearing-position correction (c) principle of a generative adversarial network (GAN) model. (d) GPT consultation using a large language model.

To provide users with real-time data visualization, automated analysis, and personalized health recommendations, we developed intelligent ECG display and processing software (Fig. 6a). The system architecture was designed to integrate a flexible ECG patch, which continuously collects ECG data, with an interpretable deep learning model for disease recognition and a generative adversarial network (GAN) model for wearing-position correction (Fig. 6b). These features are essential for the early detection and management of cardiac conditions.

We implemented four main interfaces within the software: Real-time Visualization, Position Correction, Health Assessment Generation, and GPT-Consultation. In Real-time Visualization, real-time ECG waveforms and key cardiac parameters, including heart rate (with 95% accuracy), heart rate variability, and minimum-maximum heart rate, are updated every 6 seconds. In Position Correction, we utilize a GAN model to correct the ECG patch’s wearing position, providing graphical feedback. A green icon indicates correct placement, while a red icon signifies an incorrect position, with corresponding icons lighting up based on the actual position (Fig. S8). In Health Assessment Generation, we generate detailed diagnostic reports based on the classification results of long-term ECG monitoring data, offering in-depth insights into the user’s cardiac health. Finally, in GPT-Consultation, a GPT-based interface provides personalized consultations and health advice, enhancing user understanding and engagement.

We evaluated the GAN-based wearing-position correction and achieved over 85% classification accuracy, significantly improving the precision of ECG signal monitoring (Fig. 6c and Fig. S16). The GAN combined with CNN and GRU networks is employed to correct the installation positions of ECG patches. The model’s classification accuracy across three specific installation positions is also validated. Specifically, the sensor-acquired ECG patch position data is encoded and mapped to a higher-dimensional space through a fully connected layer. A one-dimensional convolutional neural network (1D-CNN) is then utilized to extract spatial relationships between different positional parameters. Given the need for real-time adjustment of ECG patch positions, a GRU-based recurrent neural network is introduced to enable rapid output. Finally, the encoded vector of the standard installation position is incorporated, and a discriminator is used to verify whether the installation position meets the error tolerance criteria. If significant deviations are detected, adjustment suggestions are generated through a fully connected layer.

By integrating a large language model, our system is capable of conducting consultations and provide diagnostic recommendations simultaneously (Video S2). The performance of our system in delivering recommendations is comparable to other large language models (GPT-3.5 in Fig. S20 and GPT-4.0 in Fig. S21). Furthermore, users can inquire about their heart conditions and receive relevant and reasonable health advice reports (Fig. 6d and Fig. S22).

The results demonstrate that our intelligent ECG software empowers users to better manage their cardiac health, promoting early detection and timely intervention. This functionality not only enhances individual health management but also alleviates the burden on healthcare systems by reducing frequent medical consultations.

## CONCLUSION

In this study, we developed a wearable, user-friendly, and intelligent ECG monitoring system that integrates a low-motion interference ECG patch with an intelligent analysis mobile application. The ECG patch features an innovative island-and-bridge structure, comprising a central circle surrounded by three smaller circles interconnected through serpentine wires. This design provides robust adhesion (7.6 kPa), a high signal-to-noise ratio (28 dB), and low strain sensitivity, evidenced by a gauge factor of 0.005. Under 100% strain, the resistance changes by only 0.5%, guaranteeing reliable ECG data collection during movement or physical activities (Fig. 2c). These structural innovations enable continuous and accurate ECG monitoring, even under dynamic conditions, thereby enhancing the system’s overall reliability and user comfort [9,17].

To achieve precise heart disease diagnosis and ensure optimal device placement, we integrated sophisticated deep learning models into the ECG monitoring system. We employed a generative adversarial network (GAN) model for wearing-position correction, achieving an accuracy of 85%, which significantly minimizes the risk of distorted ECG signals due to improper patch placement (Fig. 2f). Additionally, our deep learning model, specifically an interpretable attention transformer network, successfully diagnosed three common heart diseases (atrial hypertrophy, myocardial infarction, and arrhythmias) with a maximum accuracy of 96% (Fig. 4c–e). The companion mobile application leverages these AI models to provide real-time visualization of ECG waveforms, heart rate, and heart rate variability, along with automatic analysis and personalized health recommendations (Fig. 6a and b). This integration of AI enhances the system’s diagnostic capabilities, enabling early detection and timely intervention for cardiac conditions [33–36].

Our intelligent ECG software offers a comprehensive suite of features that improve user experience and expand healthcare accessibility. The software provides real-time visualization of ECG data, enabling users to continuously monitor their heart health (Fig. 6a). The automatic analysis performed by the IAT and GAN models ensures accurate diagnosis and optimal device placement without the need for medical expertise. Furthermore, the GPT-based consultation interface delivers personalized clinical decision-making [37,38], including dietary recommendations, exercise guidance, and medical suggestions, within 1 second of user queries (Fig. 6d and Figs S16–S18). This real-time, interactive medical consultation feature empowers users to manage their cardiac health proactively and reduces the dependence on frequent hospital visits, thereby alleviating the burden on healthcare systems [27,29].

Our wearable ECG monitoring system demonstrates significant advancements in continuous monitoring, accurate diagnosis, and user accessibility; however, several areas warrant further exploration and improvement. (1) Enhancing deep learning model accuracy: to increase the accuracy and reliability of our predictive models, there is a need for more diverse and extensive datasets, encompassing various demographic groups and stages of heart disease [39–41]. This will ensure that the models generalize well across different populations and clinical scenarios [42–44]. (2) Multi-sensor and heterogeneous techniques integration for comprehensive health assessment: integrating more and thinner sensors, such as blood pressure monitors, electroencephalograms, accelerometers, thermal sensors, and oxygen saturation monitors, can provide a more holistic assessment of heart health [45–47]. This multi-modal approach will enable comprehensive health monitoring and more accurate diagnosis of complex cardiac conditions [48–51]. (3) Improving battery life and durability: enhancing the system’s battery life and durability is crucial for extending usage time and ensuring reliable long-term monitoring [52–54]. Exploring wireless power supply methods or energy-harvesting technologies could facilitate continuous operation without frequent recharging, thereby improving user convenience and device reliability [55–57]. Future research will focus on these aspects to refine and expand the capabilities of our ECG monitoring system. By addressing these challenges, we aim to further enhance patient outcomes and healthcare efficiency, making advanced cardiac care more accessible and effective.

## ETHICAL DECLARATION

Consent was obtained to publish identifiable images of study participants. All data were collected with the informed consent of each participant.

## Supplementary Material

nwaf425_Supplemental_Files
