# Supplementary material for: AI-enhanced flexible ECG patch for accurate heart disease diagnosis, optimal wear positioning, and interactive medical consultation
Source: Natl Sci Rev. 2025 Oct 11;12(12):nwaf425. doi: 10.1093/nsr/nwaf425 (PMC12636515; doi:10.1093/nsr/nwaf425)
Supplement: nwaf425_Supplemental_Files [file nwaf425_supplemental_files.zip › Supplementary data.pdf]

Supporting Information

AI-enhanced flexible ECG patch for accurate heart disease diagnosis, optimal wear positioning, and interactive medical consultation

Xiaojiang Huang[1,2], Youlin Yuan[3], Jiming Liu[5], Jun He[4], Yunxiang Shi[1], Shuai Gao[1], Jun Wu[1], Xingjie Xu[1], Huiqing Zhang[1,6*], Peng Li[1,*], Yao Yao[1,2,*] and Wei Huang[1,*]

[1]State Key Laboratory of Flexible Electronics (LoFE) & Institute of Flexible Electronics (IFE), Northwestern Polytechnical University, Xi'an 710072, China;

[2]School of Mechanics, Civil Engineering and Architecture, Northwestern Polytechnical University, Xi'an 710072, China;

[3]Queen Mary University of London Engineering School, Northwestern Polytechnical University, Xi'an 710072, China;

[4]China Ordnance Industry Group-machine-environment Key Laboratory, Institute for Hygiene of Ordnance Industry, Xi’an 710065, China;

[5]School of Mechanical Engineering, Xi'an Jiaotong University, Xi'an 710049, China;

[6]Sanhang Institute for Brain Science and Technology, School of Medical Research, Northwestern Polytechnical University, Xi'an 710072, China

***Corresponding authors**. E-mails: vc@nwpu.edu.cn; yaoy@nwpu.edu.cn; iampli@nwpu.edu.cn; iamhqzhang@nwpu.edu.cn

Table of Content

Supplementary Text

Figure S1. FPCB layout diagram.

Figure S2. Performance characterization of commercial electrodes.

Figure S3. Adhesion performance of our electrode to the skin with a 500 g weight.

Figure S4. Stress-strain diagrams of tensile.

Figure S5. Mechanical tensile test of resistance changes as a function of strain.

Figure S6. Stress-strain diagrams.

Figure S7. Reliable Bluetooth signal strength.

Figure S8. Wearing-position correction of the ECG patch.

Figure S9. ECG signal monitoring performance of the electrode under stretching and twisting.

Figure S10. ECG signal comparison of the electrode under stretching and twisting.

Figure S11. Heatmap of ECG (a) Pearson similarity matrix and (b) Euclidean similarity matrix between across different angles.

Figure S12. Heatmap of ECG (a) Pearson similarity matrix and (b) Euclidean similarity matrix between different placements and heart disease.

Figure S13. Performance comparison diagram with (a) LSTM and (b) Bi-LSTM models.

Figure S14. Long-term household ECG monitoring.

Figure S15. Mobile phone interface.

Figure S16. The accuracy of position correction.

Figure S17. Health assessment for myocardial infarction.

Figure S18. Health assessment for atrial enlargement.

Figure S19. Health assessment for arrhythmia.

Figure S20. Consultation based on the GPT-3.5 model.

Figure S21. Consultation based on the GPT-4.0 model.

Figure S22. Medical consultation in question-and-answer style based on the DeepSeek model.

Video S1 (separate file). Flexible ECG patch for accurate heart disease diagnosis, optimal wear positioning.

Video S2 (separate file). Interactive medical consultation.

Supplementary Text

METHODS

ECG patch fabrication for wireless electrocardiogram data collection, processing and analysis: The flexible ECG patch is a multi-layered device designed for efficient wireless ECG data collection, processing and analysis. The top encapsulation layer made of polydimethylsiloxane (PDMS) with a thickness of 1 mm. Beneath this layer lies a PI-based circuit layer, which incorporates various electronic components, a double-layered flexible printed circuit board (FPCB) with a copper thickness of 18 μm and a PI thickness of 75 μm, and a battery. The FPCB design was built using JLCPCB EDA software, and the fabrication was carried out by JLCPCB Company. The flexible ECG patch measures 5.5 cm x 5.0 cm and has three circular ECG electrodes, each with a diameter of 1 cm, connected to the core circuit area (a diameter of 3 cm) via a serpentine circuit. The serpentine circuit has a PI width of 1.5 mm and a Cu width of 0.2 mm. Key components of the circuit include a microcontroller chip (nRF52832, from Nordic Semiconductor), an analog front-end (AFE) chip (KS1081, from Kingsense), and a power management chip (LP3986, from Low Power Semi). The KS1081 AFE chip is a single-channel system designed to work with the three ECG electrodes. The electrode connectors are soldered to the ECG patch and are designed to be used with disposable ECG electrodes.The electrode back has red/black markers to aid proper user orientation. These disposable electrodes can be snapped onto the electrode connectors and carefully attached to the skin near the heart to ensure precise positioning with a high signal-to-noise ratio. The microcontroller collects the analog output from the KS1081 chip and transmits the data wirelessly via Bluetooth to a mobile application for data acquisition and analysis. The commercial ECG device used in this study is the PC80B Holter monitor (Medrui Co.). To evaluate the ECG monitoring performance of the flexible ECG patch, the signal-to-noise ratio (SNR) was calculated using the following equation:

$$\mathrm{SNR}=10\lg(P_{\mathrm{signal}}/P_{\mathrm{noise}}) \quad (1)$$

where P_{signal} and P_{noise} are the power of signal and noise, respectively. For signal

quality validation, the commercial device used was the PC80B Holter monitor (Medrui Co.). Testing scenarios: 30-minute resting recording (sitting, minimal movement); 10-minute treadmill exercise at 5 km/h (moderate motion).

Electrode Characterization Protocols： Used a CHI760E electrochemical workstation (three-electrode system: working electrode = test electrode, counter electrode = Pt sheet, reference electrode = Ag/AgCl). Test conditions: frequency range 1 Hz–10 kHz, potential 0–0.05 V (vs. Ag/AgCl). Data were fitted with the Debye model using EIS Spectrum Analyzer software. Commercial Ag/AgCl electrodes (diameter 10 mm) served as controls.Adhesion Testing: To simulate real-world skin adhesion under static and dynamic forces, we used a weight-mounting method.Motion Artifact Simulation: To mimic real - life motion artifacts (e.g., daily activities, subtle body movements), we designed test scenarios: Subtle Motion (Lab - controlled): Volunteers wore the electrode on the chest (V5 lead position) and performed standardized low-intensity movement (e.g., slow arm raising, gentle torso twisting, 5°–10° displacement, 0.5 Hz frequency).Long-term Stability & Sweat Resistance:Electrodes were applied to porcine skin (in vitro, controlled humidity/temperature: 40% RH, 25°C) for 4 days. Daily EIS measurements and ECG signal recordings (simulated via Ag/AgCl current injection) were performed. Tested during 30-minute treadmill exercise (5 km/h) to induce natural sweating.

AI for heart diseases diagnosis, wearing-position correction and consultation: For heart diseases diagnosis, an interpretable Attention Transformer (IAT) model is proposed to diagnose heart diseases. This model is trained using MATLAB 2024. After training, the IAT model is converted into the Open Neural Network Exchange (ONNX) format, ensuring efficient deployment and integration within the mobile application.

- AI for wearing-position correction: To ensure the correct placement of the ECG patch, a Generative Adversarial Network (GAN) model was trained in MATLAB 2024 and then saved as ONNX file for subsequent invocation.

- AI for Consultation: The app integrates a large language model from DeepSeek-V3, which provides personalized health advice based on the recognition results of heart diseases through DeepSeek API.

Software interface and backend design: The mobile application is developed using the Qt Framework 6.3. The graphical user interface for the application was developed using graphical programming, while the backend algorithms were implemented in C++. Integration of various AI models includes the Interpretable Attention Transformer (IAT) model and the Generative Adversarial Network (GAN) model in ONNX format, and the large language model API from DeepSeek-V3. The application features an interface display, data storage, analysis capabilities, and built-in algorithms for time-frequency and entropy analysis of ECG data. Model performance is evaluated using the key metric, applied to disease diagnosis, position correction, and heart rate calculation tasks. The formula for accuracy is defined as:

$$\text{Accuracy} = \frac{TP+TN}{TP+TN+FP+FN} \tag{2}$$

where TP (True Positive) represents the number of correctly identified positive cases, TN (True Negative) represents the correctly identified negative cases, FP (False Positive) represents the incorrectly identified positive cases, and FN (False Negative) represents the missed positive cases. Accuracy reflects the overall correctness of the model's predictions and serves as a crucial performance indicator.

Mechanical experiments and simulations: Uniaxial tensile tests were conducted using a material mechanics testing machine (INSTRON 3344, INSTRON). The moving speed of the testing machine was set to 2 mm s^{-1}. The sample for uniaxial tensile tests was the serpentine structure of the circuit board, with dimensions of PI width of 1.5 mm and Cu width of 0.2 mm. In its relaxed states, the length of the serpentine structure was 4 cm. To measure the resistance of the serpentine structure, Cu wires were soldered on two sides of the serpentine structure. The strain sensitivity was defined as the following equation:

$GF=(\Delta R/R_0)/\varepsilon$ (3)

where R_0 and R represent the initial resistance and real-time resistance change, respectively, while ε denotes the applied strain. The adhesion strength of disposable ECG electrodes is tested using the INSTRON 3344 materials testing machine with 180° peel test fixtures. The electrode is attached to cleaned and dried skin and pressed with 0.1 kg s^{-1}. The machine performs the peel test at a tensile speed of 60 mm min^{-1}, recording peel force until the electrode detaches. The adhesion strength is calculated using the following equation:

Adhesion strength $=F_a/A_a$ (4)

where F_a represents the adhesive force, and A_a represents the adhesion area.

FEA was conducted using Abaqus2022/CAE, a comprehensive software for finite element analysis. The 3D models were constructed from JLCPCB EDA, a design tool for flexible printed circuit boards. Electronic components were integrated into the PI substrate, representing the actual configuration of the ECG patch. To simplify the analysis, the influence of middle Cu circuits was excluded, focusing on the primary structural elements. The material parameters for the electronic components were assumed to be equivalent to those of the PI substrate. The entire model was parameterized for calculations using Young's modulus (E) = 2 GPa, Poisson's ratio (v) = 0.3, and density (ρ) = 1.35. All objects were meshed into linear tet elements. Mechanical analyses were performed on the serpentine circuit under different boundary and loading conditions: All objects were meshed into linear tet elements. Mechanical analyses were performed on the serpentine circuit under different boundary and loading conditions: tension (fixing *the central circular island* and applying a displacement *at the electrode center parallel to the serpentine circuit* , 5 cm), bending (applying a downward displacement at the electrode center, 5 cm), and torsion (applying a rotational displacement at the electrode center, π rad). This comprehensive approach ensured that the FEA provided valuable insights into the mechanical behavior of the ECG patch under various loading conditions, validating its design for durability and reliability.

On-body evaluation of wearable ECG patch: The flexible ECG patch was placed on the clean chest to ensure optimal contact with the skin. After installing the mobile application, Bluetooth on the smartphone was activated and paired with the ECG patch according to the app's instructions. Subsequently, the "Start monitoring" button in the software was triggered to initiate the ECG recording process. The software then automatically corrected the wearing-position to ensure accurate data collection and diagnosis. Once the data recording completed, the ECG patch was gently detached.

Participants were recruited from Zhenyuan County Traditional Chinese Medicine Hospital, comprising 52 individuals (27 males and 25 females) aged between 18 and 75 years. The participants were further categorized into three age groups: 20 young adults (18-35 years), 15 middle-aged adults (36-55 years), and 17 older adults (56-75 years). They were also classified into four health categories: 20 had normal ECG, 18 had arrhythmias, 8 had myocardial infarction, and 6 had atrial hypertrophy.

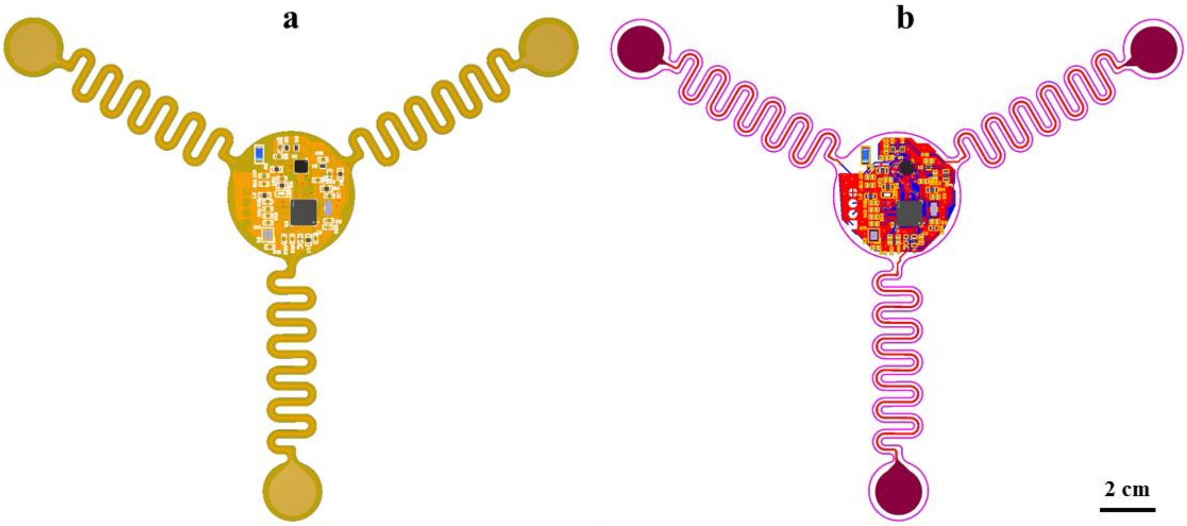

Figure S1. FPCB layout diagram.

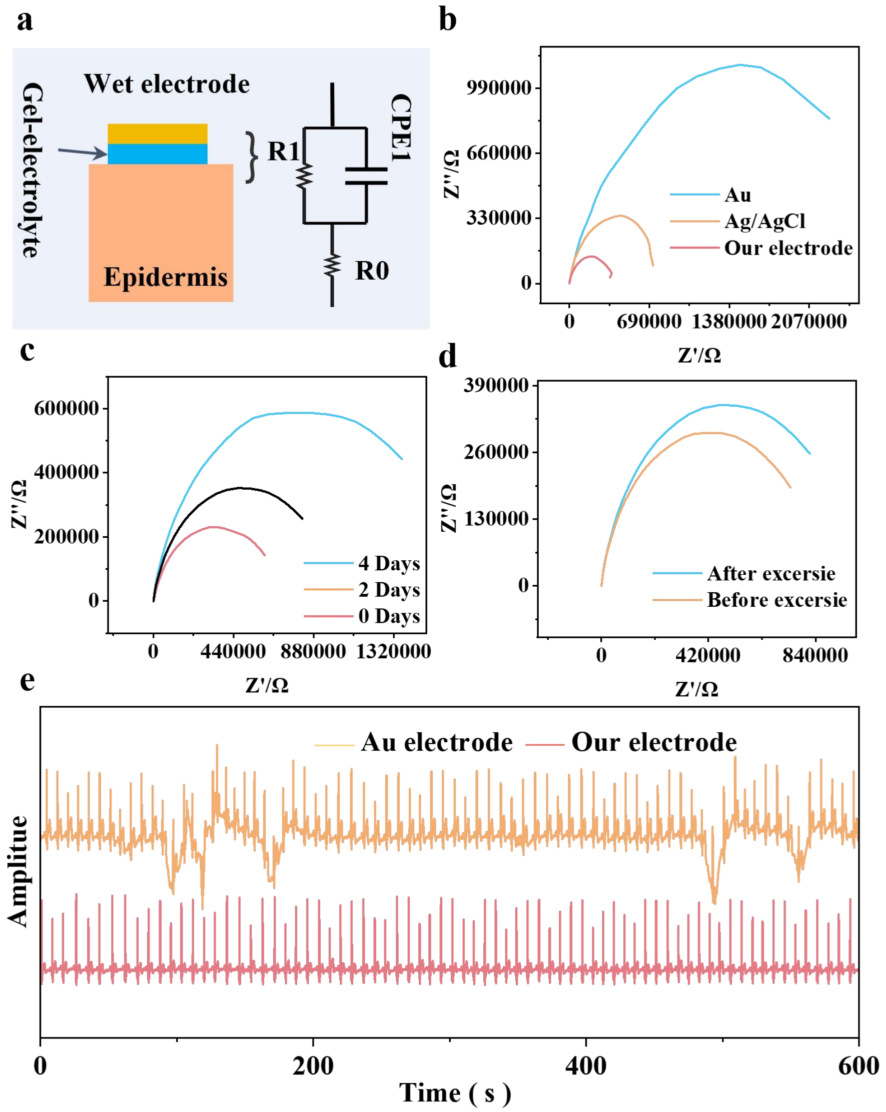

Figure S2. Performance characterization of commercial electrodes. (a) Interface impedance model, R1: interface resistance, R0: base interface resistance, cpe1: interface capacitance. (b) The interface impedance of the electrode we used compared to the Ag/AgCl dry electrode and the Au dry electrode. (c) Change in interface impedance of our electrode over time at room temperature. (d) Interface impedance before and after motion. (e) Comparison of motion artifact suppression performance between our electrode and the Au dry electrode.

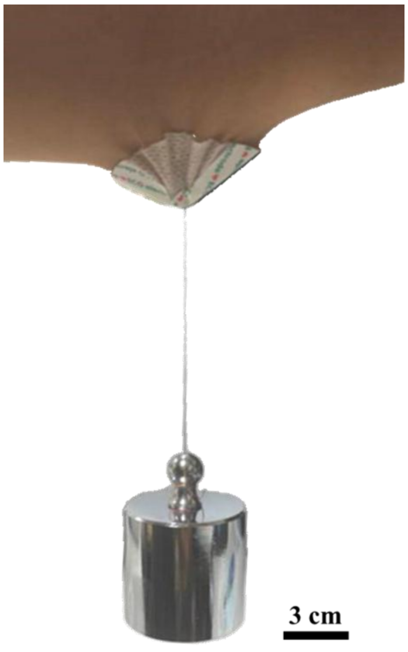

Figure S3. Adhesion performance of our electrode to the skin with a 500 g weight.

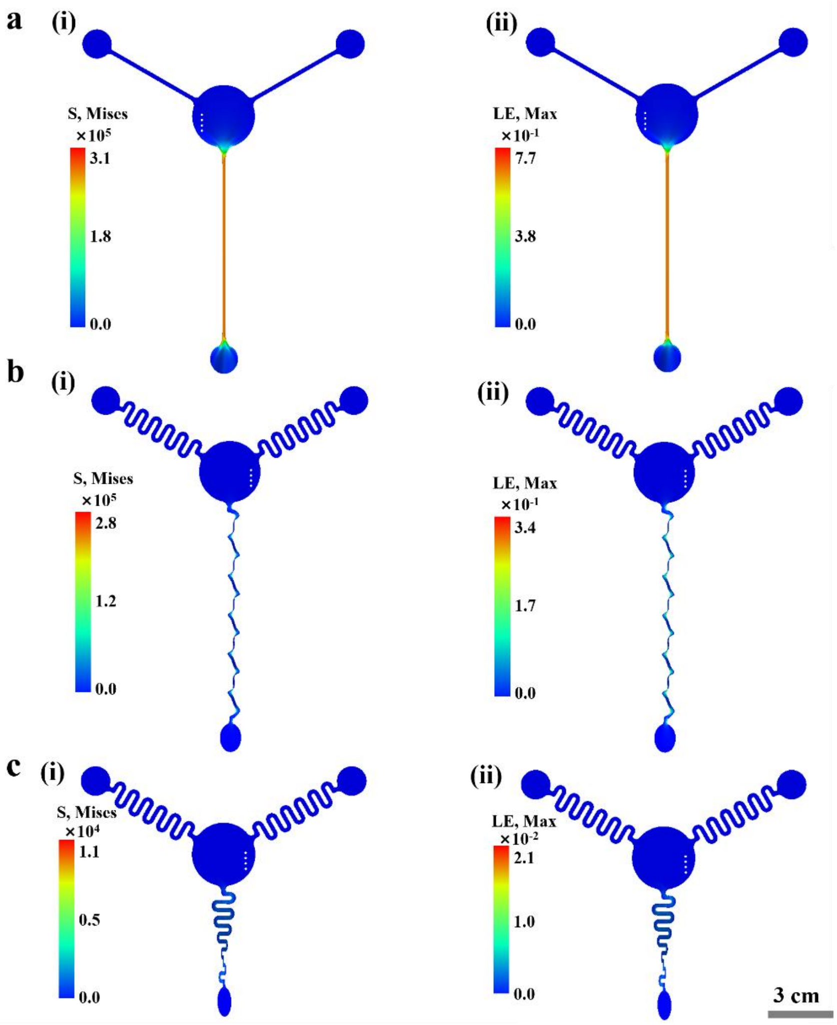

Figure S4. Stress-strain diagrams of tensile (100%) for (a) linear structure, and stress-strain diagrams of serpentine line (b) tensile (100%) and (c) torsion (180°).

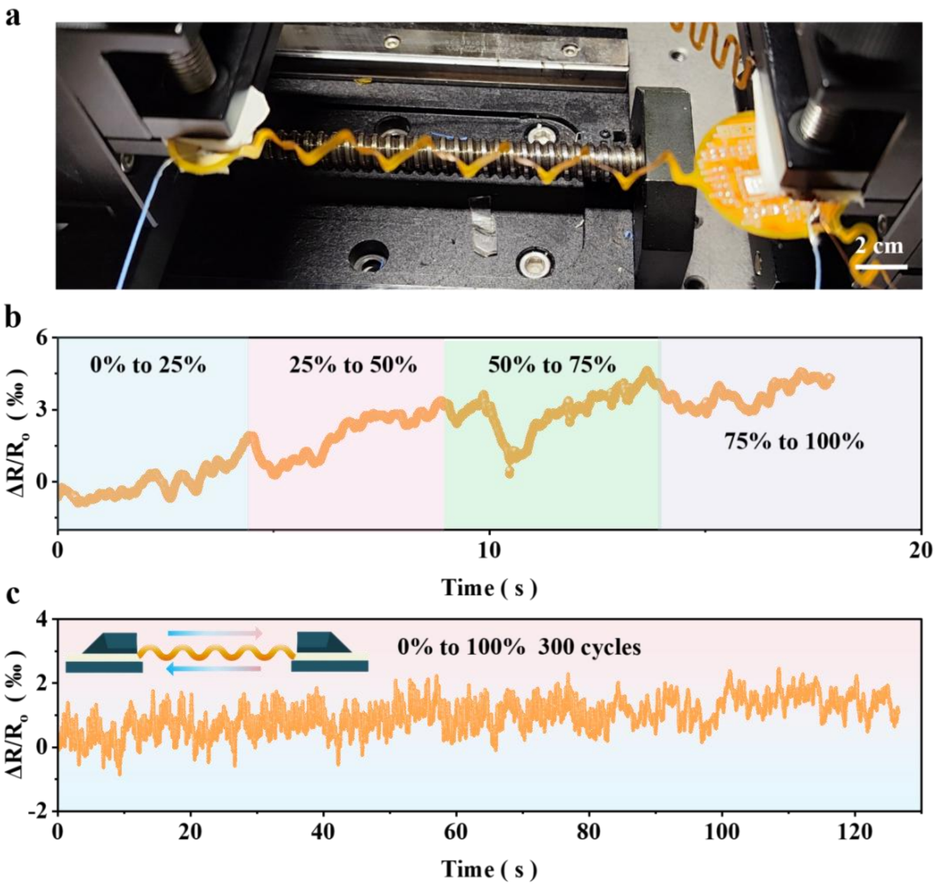

Figure S5. Mechanical tensile test of resistance changes as a function of strain. (a) Experimental setup. (b) Single-cycle tensile test. (c) Cyclic tensile test.

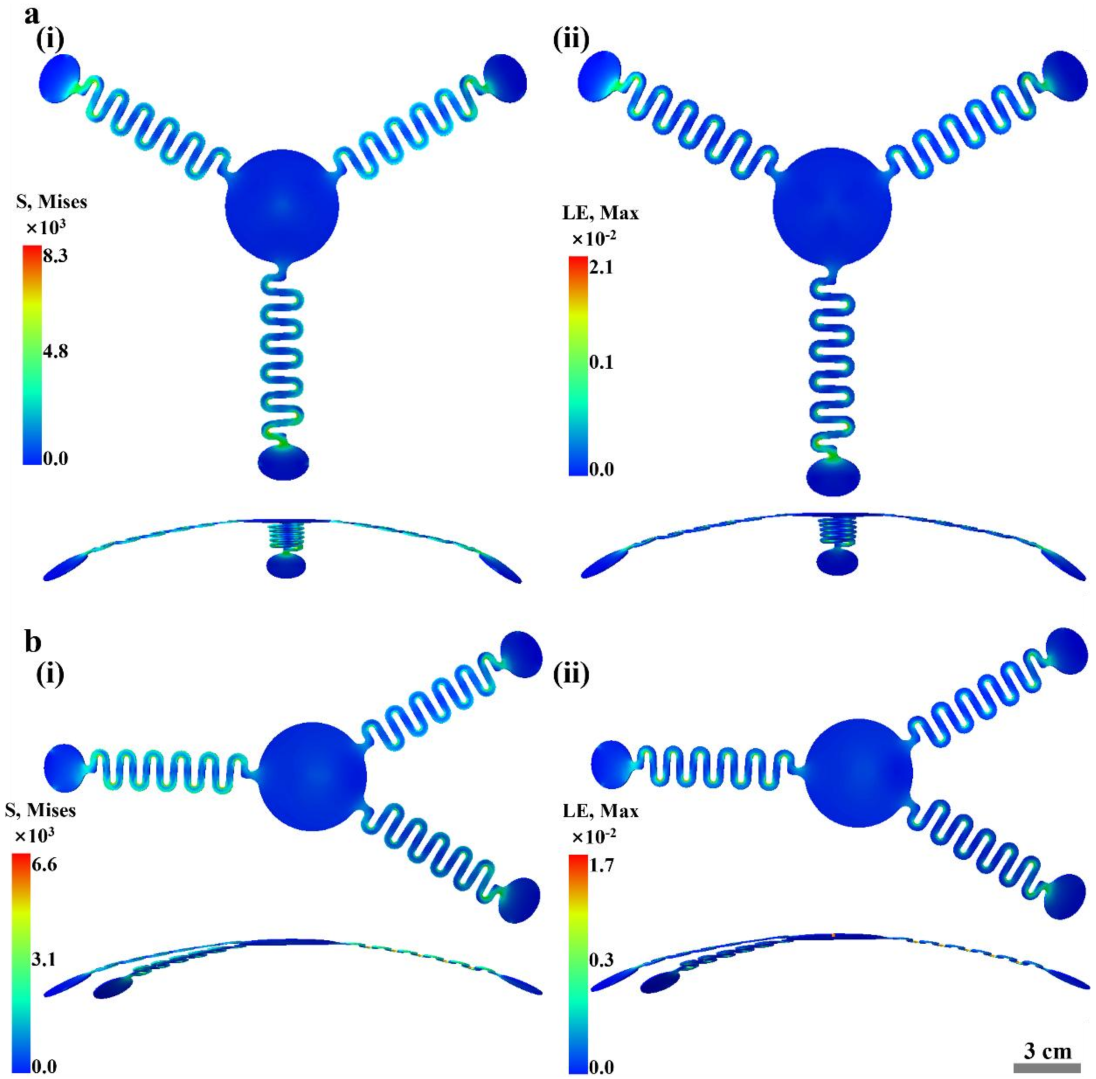

Figure S6. Stress-strain diagrams of (a) equilateral and (b) non-equilateral triangular layouts under conditions like thoracic curvature (0.05 cm^{-1}).

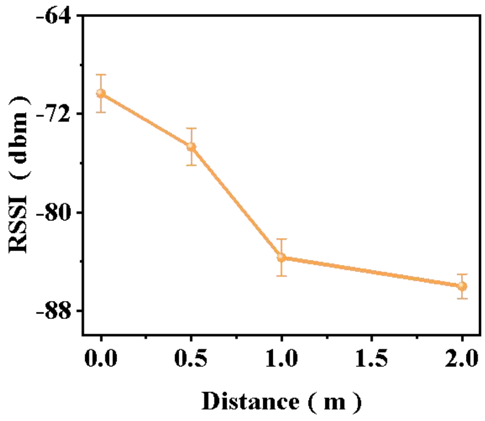

Figure S7. Reliable Bluetooth signal strength.

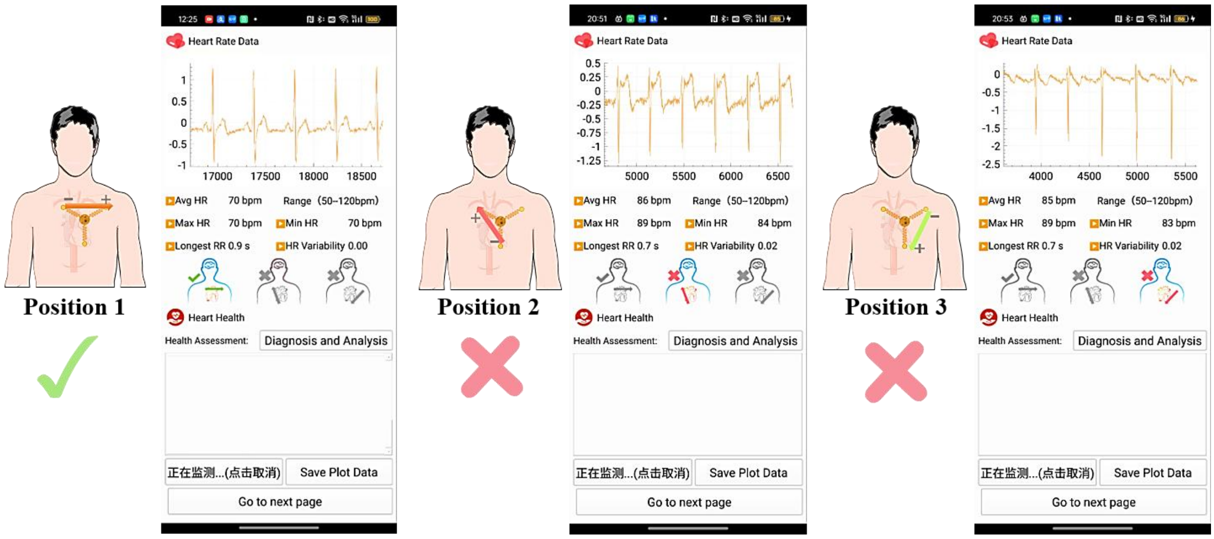

Figure S8. Wearing-position correction of the ECG patch.

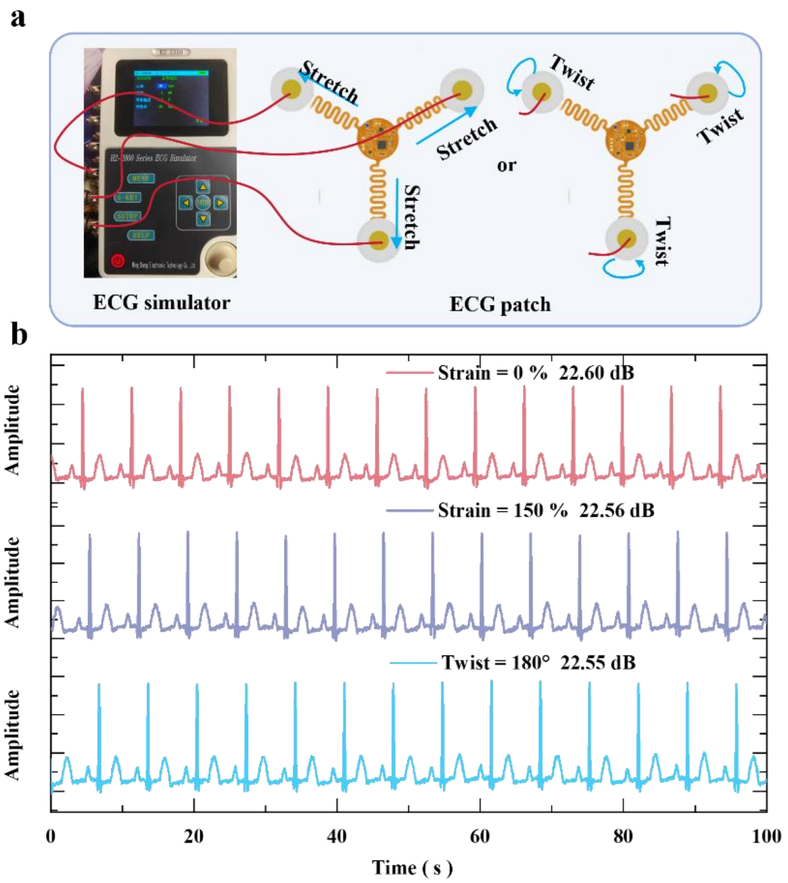

Figure S9. ECG signal monitoring performance of the electrode under stretching and twisting. (a) Experimental testing of the prototype. (b) ECG monitoring performance under tension and torsion.

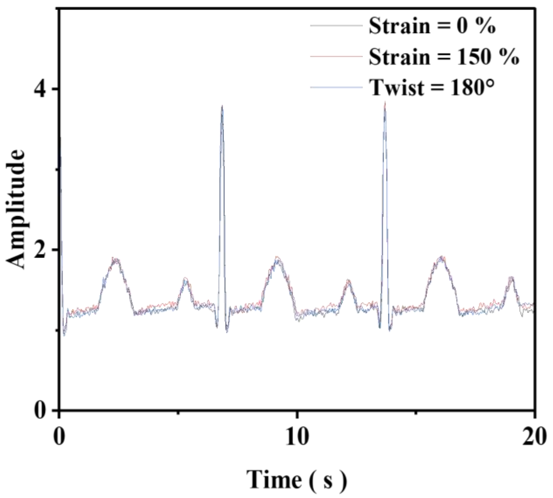

Figure S10. ECG Signal comparison of the electrode under stretching and twisting.

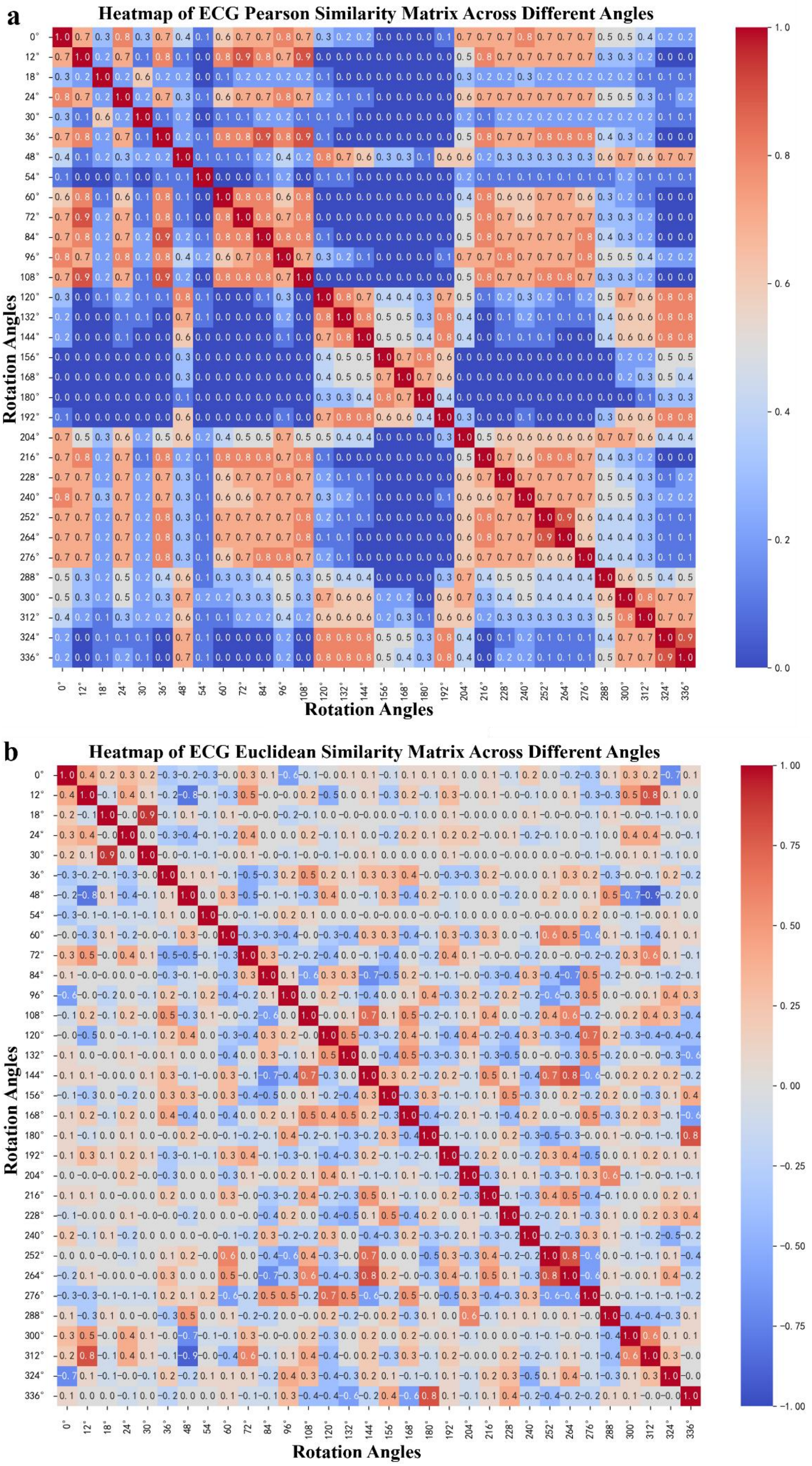

Figure S11. Heatmap of ECG (a) Pearson similarity matrix and (b) Euclidean similarity matrix between across different angles.

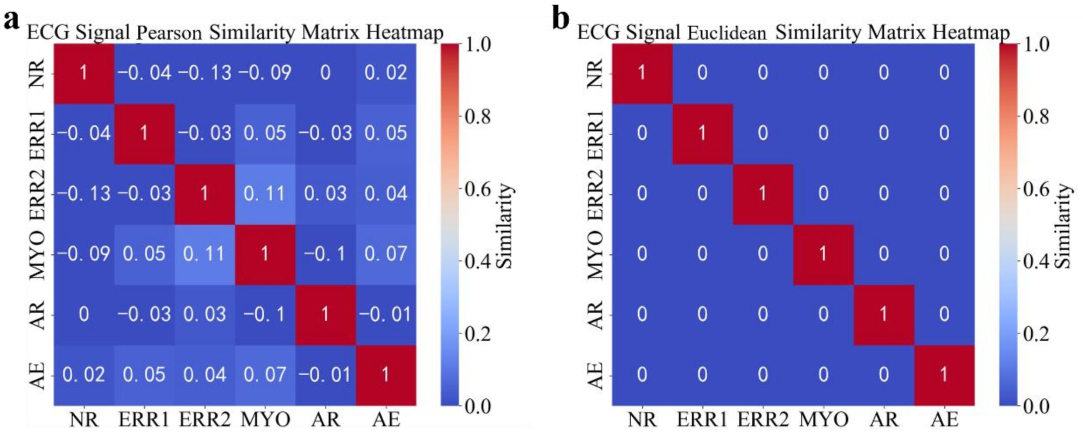

Figure S12: Heatmap of ECG (a) Pearson similarity matrix and (b) Euclidean similarity matrix between different placements and heart disease.

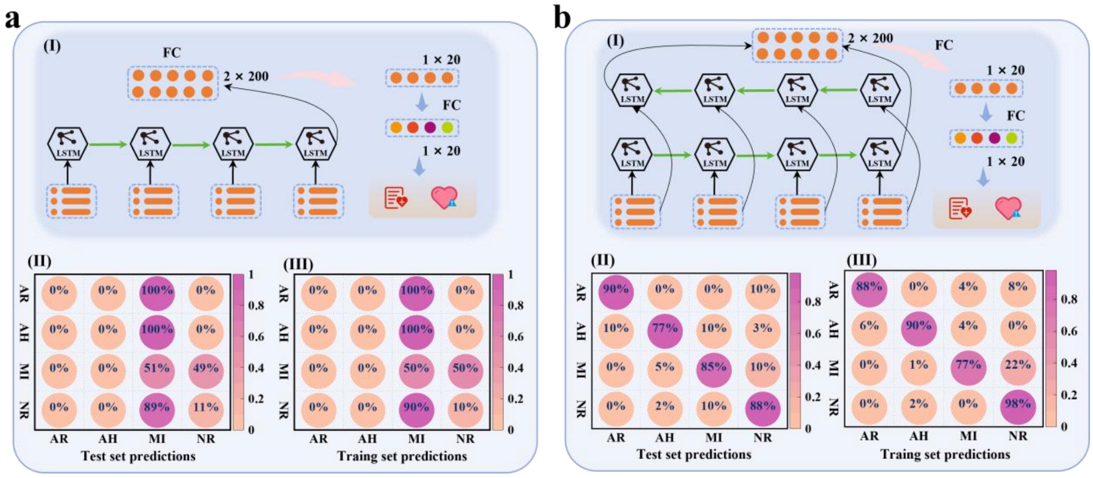

Figure S13. Performance comparison diagram with (a) LSTM and (b) Bi-LSTM models.

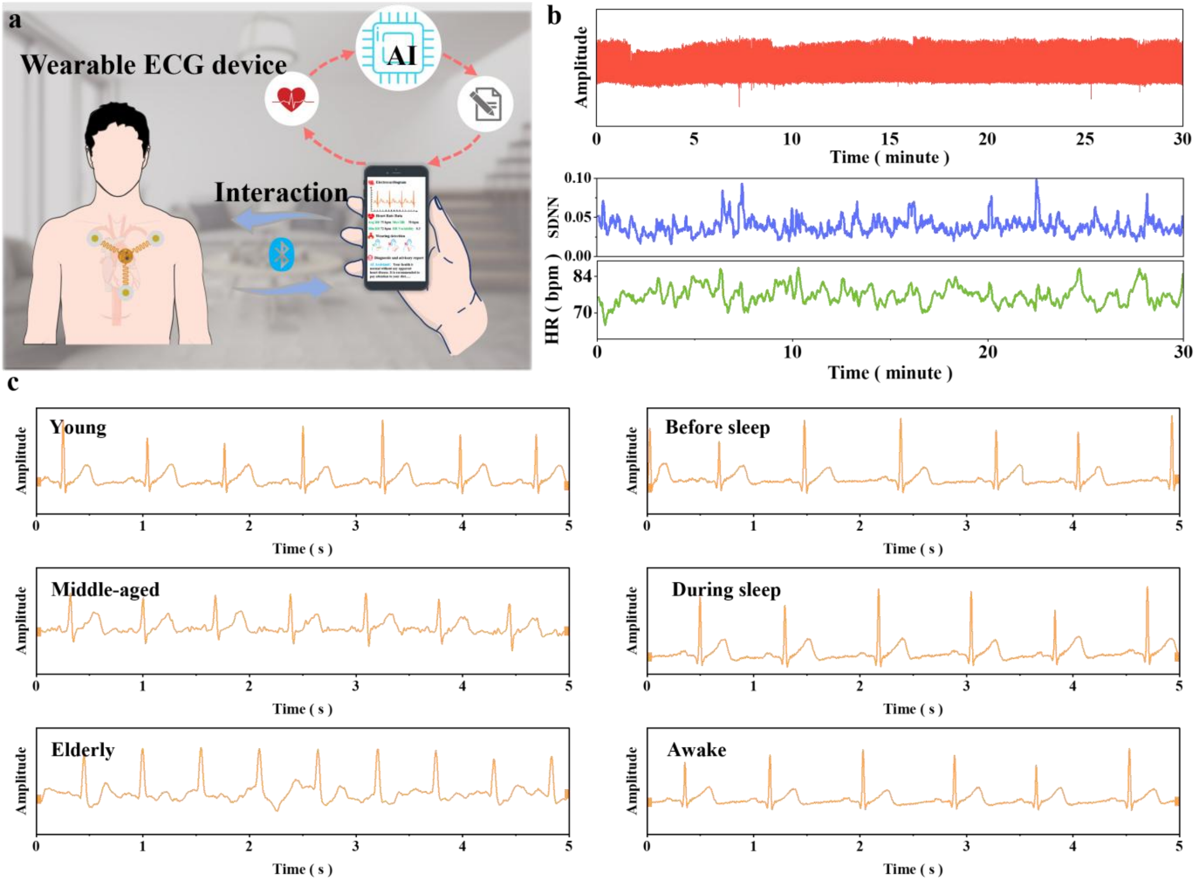

Figure S14. Long-term household ECG monitoring. (a) Schematic of household ECG monitoring. Long-term monitoring of (b) ECG, HR, and HRV. (c) ECG for different groups of people and adults at various stages of life.

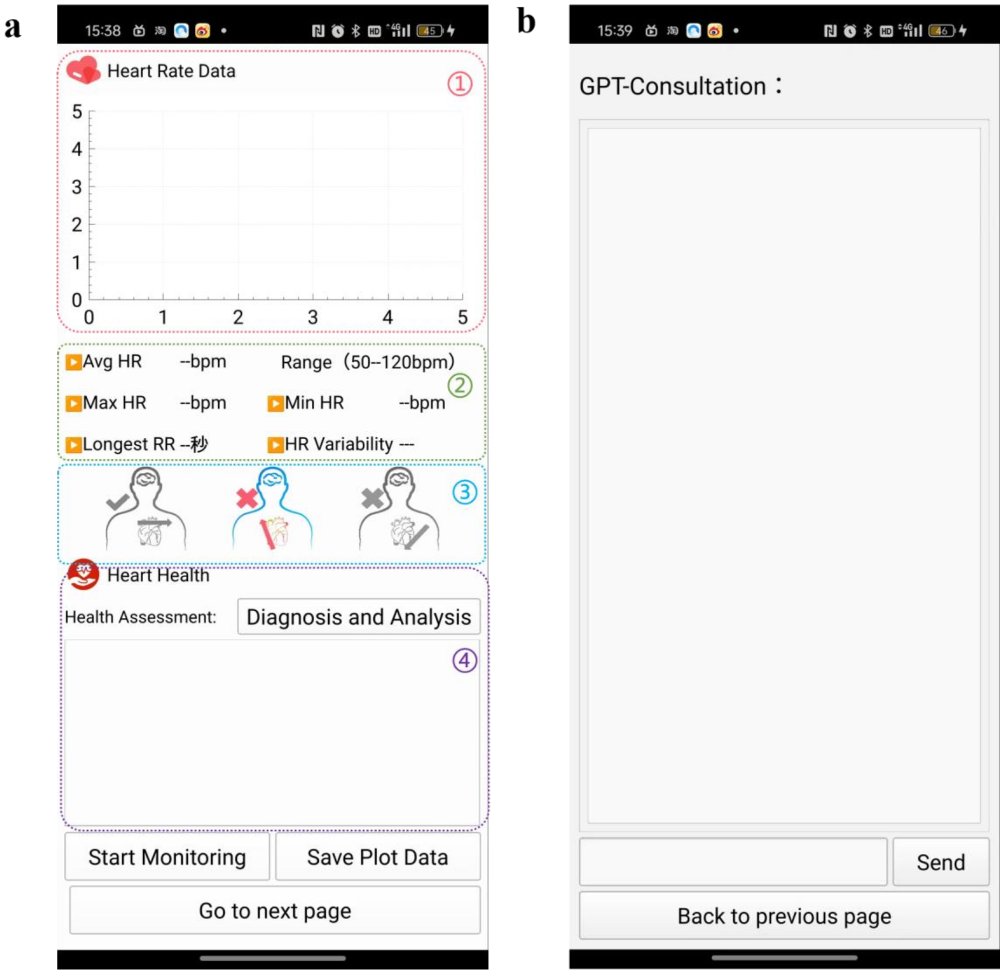

Figure S15. Mobile phone interface. (a) Main interface including ① real-time ECG display, ② heart rate data, ③ wearing-position correction, ④ health assessment. (b) GPT consultation interface.

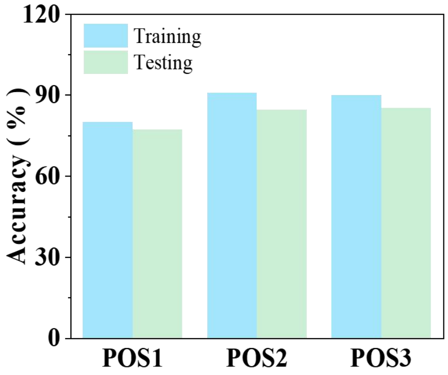

Figure S16. The accuracy of position correction.

DeepSeek

I'm sorry to hear about your diagnosis of myocardial infarction (MI), commonly known as a heart attack. This is a serious condition that requires immediate and ongoing medical attention. Here's a breakdown of what you need to know and what steps you should take:

Understanding Myocardial Infarction (MI):
1. **Cause**: MI occurs when the blood flow to a part of the heart muscle is blocked, usually due to a blood clot in a coronary artery. This blockage prevents oxygen-rich blood from reaching the heart muscle, leading to damage or death of that part of the muscle.
2. **Symptoms**: Common symptoms include chest pain or discomfort, shortness of breath, pain in the arm, neck, jaw, or back, nausea, and sweating. However, some people may experience atypical symptoms or no symptoms at all.

Immediate Actions:
1. **Seek Emergency Medical Care**: If you suspect you are having a heart attack, call emergency services immediately. Time is critical in preventing further damage to the heart muscle.
2. **Medications**: You may be given medications such as aspirin to reduce blood clotting, nitroglycerin to relieve chest pain, and anticoagulants or antiplatelet drugs to prevent further clot formation.

Long-Term Management:
1. **Cardiac Rehabilitation**: This program includes exercise training, education on heart-healthy living, and counseling to reduce stress and help you return to an active life.
2. **Medication**: You will likely need to take medications long-term to manage your condition. These may include:
  - **Statins**: To lower cholesterol levels.
  - **Beta-blockers**: To reduce heart rate and blood pressure.
  - **ACE Inhibitors or ARBs**: To help the heart pump more efficiently.
  - **Aspirin or other antiplatelet drugs**: To prevent blood clots.
3. **Lifestyle Changes**:
  - **Diet**: Adopt a heart-healthy diet low in saturated fats, trans fats, cholesterol, sodium, and added sugars. Focus on fruits, vegetables, whole grains, lean proteins, and healthy fats.
  - **Exercise**: Engage in regular physical activity as recommended by your healthcare provider.
  - **Smoking Cessation**: If you smoke, quitting is crucial for heart health.
  - **Weight Management**: Maintain a healthy weight to reduce the strain on your heart.
  - **Stress Management**: Practice stress-reducing techniques such as meditation, yoga, or deep breathing exercises.

Follow-Up Care:
1. **Regular Check-Ups**: Schedule regular follow-up appointments with your cardiologist to monitor your heart function and adjust your treatment plan as needed.
2. **Monitoring**: Keep track of your symptoms and report any changes to your healthcare provider promptly.

Psychological Support:
1. **Counseling**: Consider seeking counseling to help cope with the emotional impact of a heart attack.
2. **Support Groups**: Joining a support group can provide valuable emotional and practical support from others who have experienced similar health challenges.

Conclusion:
A myocardial infarction is a serious condition, but with proper medical care, lifestyle changes, and ongoing management, many people can lead full and active lives. It's essential to follow your healthcare provider's recommendations and take an active role in your recovery and long-term heart health.

If you have any specific questions or concerns, please don't hesitate to ask. Your health is a priority, and we are here to support you through this journey.

Figure S17. Health assessment for myocardial infarction.

DeepSeek

Based on your diagnosis of atrial enlargement (also known as atrial hypertrophy or atrial enlargement), it's important to understand the implications and necessary steps for managing this condition.

Analysis of Atrial Enlargement:
1. **Definition**: Atrial enlargement refers to the enlargement of the upper chambers of the heart (atria). This can occur in both the left and right atria, but left atrial enlargement is more common.
2. **Causes**:
  - **Hypertension (High Blood Pressure)**: Long-term high blood pressure can lead to increased workload on the heart, causing the atria to enlarge.
  - **Valvular Heart Disease**: Conditions like mitral valve stenosis or regurgitation can cause the left atrium to enlarge.
  - **Atrial Fibrillation**: This common heart rhythm disorder can lead to atrial enlargement over time.
  - **Cardiomyopathy**: Diseases of the heart muscle can also cause atrial enlargement.
  - **Chronic Lung Disease**: Conditions like COPD can lead to right atrial enlargement.

3. **Symptoms**:
  - **Shortness of Breath**: Especially during physical activity.
  - **Fatigue**: Due to reduced efficiency of the heart.
  - **Palpitations**: Irregular or rapid heartbeats.
  - **Swelling in the Legs and Ankles**: A sign of fluid retention.
  - **Chest Pain or Discomfort**: Less common but possible.

Recommendations for Management:
1. **Medical Treatment**:
  - **Blood Pressure Control**: If hypertension is a contributing factor, medications to lower blood pressure are essential.
  - **Heart Rhythm Control**: If atrial fibrillation is present, medications or procedures to restore and maintain a normal rhythm may be necessary.
  - **Diuretics**: To manage fluid retention and reduce the workload on the heart.
  - **Anticoagulants**: If atrial fibrillation is present, to prevent blood clots and reduce the risk of stroke.

2. **Lifestyle Modifications**:
  - **Diet**: A heart-healthy diet low in salt, fat, and cholesterol is recommended.
  - **Exercise**: Regular, moderate exercise can help improve heart function and overall health.
  - **Weight Management**: Maintaining a healthy weight can reduce the strain on the heart.
  - **Avoiding Stimulants**: Caffeine and nicotine can exacerbate heart rhythm disturbances.

3. **Monitoring and Follow-Up**:
  - **Regular Check-Ups**: Regular visits to your healthcare provider to monitor blood pressure, heart function, and any changes in symptoms.
  - **Echocardiograms**: Periodic echocardiograms to assess the size and function of the atria and overall heart function.
  - **Holter Monitor**: If arrhythmias are suspected, a Holter monitor may be used to record heart activity over 24-48 hours.

4. **Surgical or Interventional Options**:
  - **Valve Repair or Replacement**: If valvular disease is the cause, surgical intervention may be necessary.
  - **Catheter Ablation**: For atrial fibrillation, catheter ablation can be considered to eliminate the abnormal electrical pathways in the heart.

Conclusion:
Atrial enlargement is a serious condition that requires careful management. By working closely with your healthcare team, you can effectively manage the condition and reduce the risk of complications. It's important to follow your treatment plan, make necessary lifestyle changes, and attend all follow-up appointments to ensure optimal heart health.

Figure S18. Health assessment for atrial enlargement

DeepSeek

Based on your diagnosis of heart arrhythmia (irregular heartbeat), it's important to understand that arrhythmias can range from minor issues that may not require immediate treatment to more serious conditions that could pose significant health risks. Here are some key points to consider:

1. **Types of Arrhythmias:**
 - **Tachycardia:** Heart rate faster than 100 beats per minute.
 - **Bradycardia:** Heart rate slower than 60 beats per minute.
 - **Atrial Fibrillation:** Irregular and often rapid heart rate, usually originating in the atria.
 - **Premature Contractions:** Early beats that disrupt the normal rhythm.

2. **Symptoms:**
 - Palpitations (feeling of skipped or extra beats).
 - Chest pain or discomfort.
 - Shortness of breath.
 - Dizziness or lightheadedness.
 - Fainting or near-fainting episodes.

3. **Risk Factors:**
 - Age (older adults are more prone).
 - Heart disease (e.g., coronary artery disease, heart failure).
 - High blood pressure.
 - Diabetes.
 - Smoking, excessive alcohol or caffeine intake.
 - Stress and anxiety.
 - Certain medications.

4. **Diagnostic Tests:**
 - **Electrocardiogram (ECG):** Records the heart's electrical activity.
 - **Holter Monitor:** A portable ECG device worn for 24-48 hours.
 - **Event Monitor:** Records heart activity during specific symptoms.
 - **Echocardiogram:** Provides images of the heart's structure and function.
 - **Stress Test:** Evaluates heart function during physical activity.

5. **Treatment Options:**
 - **Medications:** Antiarrhythmics to regulate heartbeat.
 - **Cardioversion:** Electrical shock to reset the heart rhythm.
 - **Catheter Ablation:** Destroys small areas of heart tissue causing irregular beats.
 - **Pacemaker or Implantable Cardioverter-Defibrillator (ICD):** Devices to regulate heart rhythm.
 - **Lifestyle Changes:** Quit smoking, limit alcohol, manage stress, maintain a healthy diet, and exercise regularly.

6. **Follow-Up:**
 - Regular check-ups with your cardiologist.
 - Monitoring symptoms and reporting any changes.
 - Adhering to prescribed treatment plans.

7. **Emergency Situations:**
 - Seek immediate medical attention if you experience severe chest pain, fainting, or difficulty breathing.

8. **Psychological Support:**
 - Consider counseling or support groups to manage stress and anxiety related to heart conditions.

Conclusion:
Heart arrhythmia is a serious condition that requires careful management. It's crucial to work closely with your healthcare provider to determine the best course of action tailored to your specific condition. Regular monitoring and lifestyle adjustments can significantly improve your quality of life and reduce the risk of complications.

If you have any specific questions or need further clarification, please don't hesitate to ask.

Figure S19. Health assessment for arrhythmia.

I have a possibility of atrial fibrillation, please give some health advice on living, diet, medication

Human

GPT-3.5

Managing atrial fibrillation (AFib) involves a combination of lifestyle changes, dietary adjustments, and medication. Here's a comprehensive guide to help you manage the condition:

1. Lifestyle Changes
(1) Exercise Regularly: Engage in regular, moderate exercise like walking or swimming, but avoid overexertion. Consult your doctor before starting any new exercise regimen.
(2) Maintain a Healthy Weight: Achieving and maintaining a healthy weight can reduce the risk of AFib episodes and improve overall cardiovascular health.
(3) Limit Alcohol: Excessive alcohol can trigger AFib. If you choose to drink, do so in moderation.
(4) Avoid Caffeine: Some people find that caffeine can trigger AFib episodes. Monitor your intake and discuss it with your doctor.
(5) Quit Smoking: Smoking can exacerbate heart problems and AFib. Seek resources and support to help you quit.

2. Diet
(1) Eat a Heart-Healthy Diet: Focus on a diet rich in fruits, vegetables, whole grains, lean proteins, and healthy fats. The Mediterranean diet is often recommended.
(2) Limit Sodium: Reducing sodium intake can help manage blood pressure and reduce fluid retention, which can be beneficial for AFib.
(3) Reduce Processed Foods: Minimize the consumption of processed foods high in sodium and unhealthy fats.
(4) Watch Your Potassium: Potassium helps regulate heart function. Eating potassium-rich foods like bananas, oranges, and spinach can be beneficial, but consult your doctor if you're on medications that affect potassium levels.
(5) Balance Omega-3 Fatty Acids: Incorporate sources of omega-3 fatty acids, like fatty fish, flaxseeds, and walnuts, which can be beneficial for heart health.

3. Medication
(1) Anticoagulants: Medications like warfarin or newer alternatives (e.g., apixaban, rivaroxaban) are used to reduce the risk of stroke. Your doctor will determine the appropriate medication and dosage.
(2) Rate-Control Medications: Drugs such as beta-blockers or calcium channel blockers may be prescribed to control the heart rate during AFib episodes.
(3) Rhythm-Control Medications: Sometimes, medications to help restore normal heart rhythm, such as flecainide or sotalol, are used.
(4) Monitor Your Medications: Regularly review your medications with your healthcare provider to ensure they are effective and adjust dosages as needed.

4. Regular Check-ups
(1) Monitor Your Condition: Regular follow-ups with your healthcare provider are crucial to monitor the effectiveness of your treatment plan and make any necessary adjustments.
(2) Check for Complications: Regular evaluations can help identify and manage potential complications, such as blood clots or heart failure.

5. Stress Management
(1) Practice Relaxation Techniques: Techniques such as deep breathing, meditation, or yoga can help manage stress, which can affect heart rhythm.

6. Emergency Plan
(1) Know When to Seek Help: Be aware of symptoms that may require immediate medical attention, such as severe chest pain, shortness of breath, or sudden dizziness.

Always consult with your healthcare provider before making significant changes to your lifestyle or starting any new treatments. They can provide personalized advice based on your specific health needs and condition.

Figure S20. Consultation based on the GPT-3.5 model.

Human

I have a possibility of atrial fibrillation, please give some health advice on living, diet, medication

GPT-4.0

If you have a possibility of atrial fibrillation (AFib), it's important to follow a comprehensive plan to manage your condition. Here are some general health tips on lifestyle, diet, and medication that might help:

1.Lifestyle

(1) Regular Monitoring:Regularly check your heart rate and blood pressure.If you have a wearable device that monitors ECG, use it to keep track of irregularities.

(2) Exercise:Engage in regular, moderate physical activity like walking, swimming, or cycling.Avoid strenuous exercises that may strain your heart, especially if not recommended by your healthcare provider.

(3) Stress Management:Practice relaxation techniques such as deep breathing, meditation, or yoga. Avoid stressful situations when possible, as stress can trigger AFib episodes.

(4) Avoid Stimulants:Limit or avoid caffeine, alcohol, and nicotine, as these can exacerbate AFib symptoms. Be cautious with over-the-counter medications that may contain stimulants.

2.Diet

(1) Heart-Healthy Diet:Follow a diet rich in fruits, vegetables, whole grains, lean proteins, and healthy fats like those found in fish and nuts. Consider the DASH (Dietary Approaches to Stop Hypertension) diet or the Mediterranean diet, both of which are heart-healthy.

(2) Reduce Sodium:Limit your intake of salt and processed foods high in sodium to help control blood pressure.

(3) Potassium-Rich Foods:Include foods high in potassium, such as bananas, oranges, spinach, and sweet potatoes, which can help regulate heart function.

(4) Omega-3 Fatty Acids:Incorporate sources of omega-3 fatty acids like fatty fish (salmon, mackerel), flaxseeds, and walnuts to support heart health.

3.Medication

(1) Adhere to Prescriptions:Take medications as prescribed by your healthcare provider. This may include blood thinners, beta-blockers, or antiarrhythmic drugs.Never stop taking your medications without consulting your doctor.

(2) Regular Check-Ups:Visit your healthcare provider regularly to monitor your condition and adjust medications as needed.

If you experience any side effects or new symptoms, inform your doctor immediately.

(3) Drug Interactions:Be aware of potential drug interactions with other medications you may be taking. Always check with your doctor or pharmacist before starting new medications, including over-the-counter drugs or supplements.

(4) Managing Risk Factors:If you have other conditions such as high blood pressure, diabetes, or sleep apnea, make sure they are well-managed as they can contribute to AFib.

4.General Advice

(1) Educate Yourself: Learn about AFib so you can make informed decisions about your health.

(2) Stay Connected: Keep in regular contact with your healthcare team and consider joining a support group for individuals with AFib.

Please consult with your healthcare provider for personalized advice and treatment options tailored to your specific situation.

Figure S21. Consultation based on the GPT-4.0 model.

Human: Hello!

DeepSeek: Hello! I'm here to help you with your heart health. Could you please tell me a bit about your current symptoms, if any? Also, your age, gender, any chronic conditions you might have, and your general lifestyle including your diet, exercise routine, and sleep patterns? This information will help me provide you with the best advice and care.

Human: Hello! Thank you for offering to help with my heart health. Here's some information about me:

1.Symptoms: Currently, I don't have any specific heart-related symptoms, but I do occasionally feel short of breath after climbing stairs.
2.Age: I'm 45 years old.
3.Gender: I identify as male.
4.Chronic Conditions: I don't have any known chronic conditions, but I was diagnosed with high blood pressure a few years ago, which is under control with medication.

General Lifestyle:
1.Diet: I try to eat a balanced diet with plenty of vegetables and lean meats. However, I do have a sweet tooth and often indulge in desserts.
2.Exercise Routine: I walk for about 30 minutes every day, and I also play tennis twice a week.
3.Sleep Patterns: I generally sleep well, averaging about 7 hours per night.
I hope this information helps. Please let me know how I can further improve my heart health or if there are any specific recommendations you have for me.

DeepSeek: Thank you for providing detailed information about your health and lifestyle. Based on what you've shared, here are some recommendations and considerations to help you maintain and potentially improve your heart health:

1.Monitor Your Blood Pressure: Since you have a history of high blood pressure, it's important to keep it under control. Ensure you're regularly checking your blood pressure and adhering to your medication regimen as prescribed by your healthcare provider.
2.Dietary Adjustments: While you're already eating a balanced diet, you might consider reducing your intake of sugary desserts to help manage your blood pressure and overall heart health. Opt for healthier dessert options like fruits or dark chocolate with a higher cocoa content.
3.Increase Physical Activity: Your current exercise routine is good, but you might benefit from increasing the intensity or duration of your workouts. Since you experience shortness of breath after climbing stairs, incorporating more cardiovascular exercises like swimming or cycling could be beneficial. Always consult with your doctor before significantly changing your exercise regimen.
4.Breathing and Relaxation Techniques: To help with the occasional shortness of breath, you might find it helpful to practice deep breathing exercises or other relaxation techniques. These can improve your lung capacity and overall respiratory health.
5.Sleep Hygiene: Since you're already getting a good amount of sleep, focus on maintaining good sleep hygiene. This includes keeping a consistent sleep schedule, creating a relaxing bedtime routine, and ensuring your sleep environment is conducive to rest.
6.Regular Check-ups: Schedule regular check-ups with your healthcare provider to monitor your heart health and adjust your treatment plan as needed. Remember, small changes in your daily habits can have a significant impact on your heart health. If you have any concerns or if your symptoms change, don't hesitate to reach out to your healthcare provider for further evaluation and guidance.

Figure S22. Medical consultation in question-and-answer style based on the DeepSeek model.
